# The IQ67‐domain protein IQD1 regulates fruit shape through complex multiprotein interactions in pepper (*Capsicum annuum* L.)

**DOI:** 10.1111/pbi.70078

**Published:** 2025-04-11

**Authors:** Lianzhen Mao, Yiyu Shen, Qingzhi Cui, Yu Huang, Xiang Zhang, Junheng Lv, Wujun Xing, Dan Zhang, Naying Fang, Daqing Chen, Zhuoxuan Wu, Peiru Li, Minghua Deng, Lijun Ou, Xuexiao Zou, Zhoubin Liu

**Affiliations:** ^1^ Engineering Research Center for Horticultural Crop Germplasm Creation and New Variety Breeding, Ministry of Education, Key Laboratory for Vegetable Biology of Hunan Province, College of Horticulture Hunan Agricultural University Changsha Hunan China; ^2^ Key Laboratory of Vegetable Biology of Yunnan Province, College of Landscape and Horticulture Yunnan Agricultural University Kunming China

**Keywords:** pepper, fruit shape, CaIQD1, natural variation, microtubule

## Abstract

Natural genetic variation can be used to improve important crop agronomic traits, and understanding the genetic basis of natural variation in fruit shape can help breeders develop pepper varieties that meet market demand. In this study, we identified a QTL controlling fruit length–width ratio by conventional genetic mapping, encoding a previously uncharacterized gene *CaIQD1*. Reduced *CaIQD1 expression* resulted in short and wide fruits in pepper, whereas heterologous overexpression of *CaIQD1* resulted in narrower fruits in tomato. Further experiments suggested that *CaIQD1* regulates fruit shape in pepper by affecting cell proliferation, expansion and morphological changes. CaIQD1 also has a direct protein interaction with CaOFP20 in CaTRM‐like‐CaOFP20. Reduced *CaOFP20 expression* caused pepper fruits to become elongated and curved, whereas reduced *CaTRM‐like* expression led to the formation of rounder fruits. These gene expression changes had a significant effect on the expression of genes related to the cell cycle and cell expansion. The CaTRM‐like‐CaOFP20‐CaIQD1 module may thus represent a conserved regulatory pathway for controlling pepper fruit shape. CaIQD1 also showed direct interactions with the pepper calmodulin CaCaM7, the tubulin CaMAP70‐2 and the microtubule motor protein CaKLCR1, suggesting that the regulation of fruit shape by CaIQD1 is related to changes in microtubule dynamics mediated by Ca^2+^‐CaM. We also found that CaIQD1 interacts with several homologues of genes that typically regulate fruit shape in other plant species. In summary, our results show that CaIQD1 acts as a core hub in regulating pepper fruit shape through interactions with multiple proteins.

## Introduction

Fruit shape is an important characteristic in crop cultivation, domestication and selection (Liu *et al*., 2023). The shape and size of fruit affect crop yield and quality, consumer consumption preferences, packaging demand and market value (Che *et al*., 2023; Yu *et al*., 2022). The fruit is the edible organ of pepper (*Capsicum annuum* L.), a vegetable crop with significant economic and biological value (Ma *et al*., 2021). Identification of the genes and molecular regulatory mechanisms that determine pepper fruit shape is important for the development of new pepper varieties with fruit shapes suitable for different market needs.

Fruit shape is typically quantified on the basis of fruit diameter (FD), fruit length (FL) and the length‐to‐width ratio (fruit shape index, FSI) (Ma *et al*., 2022). Most studies of fruit shape‐related genes and regulatory pathways in Solanaceae crops have focused on tomato, and differences in tomato fruit shape are largely explained by the ovate family proteins (OFPs), *fruit shape chr 8.1* (*fs8.1*), *SUN* and *GLOBE* (Rodríguez *et al*., 2011; Sierra‐Orozco *et al*., 2021; Zhu *et al*., 2023). OVATE and OFP20 can interact with the tonneau1 recruitment motif 5 (TRM5) to form a TRM‐OFP complex, which regulates tomato fruit shape by regulating the cell division pattern in the fruit (Zhang *et al*., 2023). In model crops tomato, rice and Arabidopsis thaliana, some plant‐specific IQ67 domain proteins (IQDs) were involved in the morphological development of plant organs, such as *SlSUN*/*SlIQD21* (Bao *et al*., 2023; Xiao *et al*., 2008), *AtIQD1*/*AtIQD2* (Bürstenbinder *et al*., 2013; Zang *et al*., 2021) and *OsIQD14*/*GW5*/*GSE5* (Duan *et al*., 2017; Liu *et al*., 2017; Yang *et al*., 2020). In general, OFPs, Tonneau1 Recruiting Motif family proteins (TRMs) and IQDs family genes have functional redundancy and diversity in regulating organ shape, and the TRM‐OFP and IQD pathways have been repeatedly reported as conservative pathways for regulating the fruit shape of tomatoes, cucumbers, rice, etc. (Li *et al*., 2023; Snouffer *et al*., 2020; Wu *et al*., 2018; Zhang *et al*., 2023), but the interaction between TRM‐OFP and IQD in regulating the shape of fleshy fruits is not clear. CaOFP20 is the only protein reported to inhibit the length of pepper fruit (Borovsky *et al*., 2022). It is still unclear whether and how other OFP, TRM and IQD regulate the fruit shape of pepper.

Microtubule (MT) and cytoskeletal dynamics also play important roles in fruit morphogenesis (Hashimoto, 2015). Ordered cortical MT arrays influence plant cell shape by controlling cellulose orientation and cell wall organization to determine the cell expansion axis (Gutierrez *et al*., 2009). The cytoskeleton (including both microfilaments and MTs) is crucial for determining cell‐plate orientation and plant organ morphology (Bao *et al*., 2024). Ca^2+^‐CAM has been reported to mediate the arrangement and reorganization of microtubules in Arabidopsis and rice (Bürstenbinder *et al*., 2013, 2017). IQDs are thought to be core factors that integrate brassinosteroid (BR)–auxin and calcium signals to regulate MT dynamics and reorientation, thereby affecting cell and organ shape (Guo *et al*., 2021; Wang *et al*., 2019). For example, IQD2 forms a complex with the microfilament‐binding protein (NET3C) and the kinesin light chain‐related (KLCR) protein to participate in the endoplasmic reticulum–plasma membrane interaction and the establishment of cytoskeletal structure, thus affecting the establishment of plant cell morphology (Zang *et al*., 2021). MAP65‐1 in microtubule‐associated proteins (MAPs) and SUN18/IQD26, the second of which also binds calmodulin, interact to regulate the direction of cell division in tomato fruits and affect fruit morphogenesis (Bao *et al*., 2024). In rice, OsIQD14 is induced by auxin and controls cell shape by regulating MT behaviour, thereby affecting grain size (Yang *et al*., 2020). Nonetheless, it remains unknown whether and how dynamic changes in the cytoskeleton affect pepper fruit morphogenesis. Likewise, the mechanism by which IQD proteins coordinately regulate MT dynamics to integrate environmental and Ca^2+^ signals for control of cell morphogenesis and plant shape remains to be clarified.

Here, we crossed two homozygous annual pepper lines with round (D39) and long conical (D40) fruit shapes obtained from the F_7_ generation of selfed materials and used the resulting segregating populations to finely map and clone a major QTL for pepper fruit shape on chromosome 10. This QTL explained 26% of the phenotypic variation in FSI in the population and contained an *IQD* family gene that we named *CaIQD1*. Functional verification experiments confirmed that *CaIQD1* regulated the aspect ratio of pepper fruit. Protein interaction experiments confirmed the interaction relationship between CaIQD1, CaOFP20 and CaTRM‐like. Changes in the expression of CaIQD1, CaOFP20 and CaTRM‐like resulted in changes in fruit shape accompanied by changes in pericarp cell number and cell division and were most likely caused by altered expression of cell cycle and cell expansion genes, although the effects of individual proteins on the expression of genes related to the cell cycle and cell expansion were slightly different. CaIQD1 also showed direct interactions with the Ca^2+^ sensor CaCAM7, as well as the MT‐associated protein CaMAP70‐2 and the MT motor protein CaKLCR1, indicating that cytoskeleton‐related proteins play an important role in fruit shape variation. In light of previous work on fruit shape regulation in other species, our findings suggest that CaIQD1 participates in CaM‐dependent regulation of MT dynamics and interacts with CaSUN, CaIQD17, CaIQD3 and CaDOF1.4 proteins, influencing pepper fruit shape through complex multiprotein interactions.

## Results

### Inheritance of pepper fruit shape

**Figure 1:**
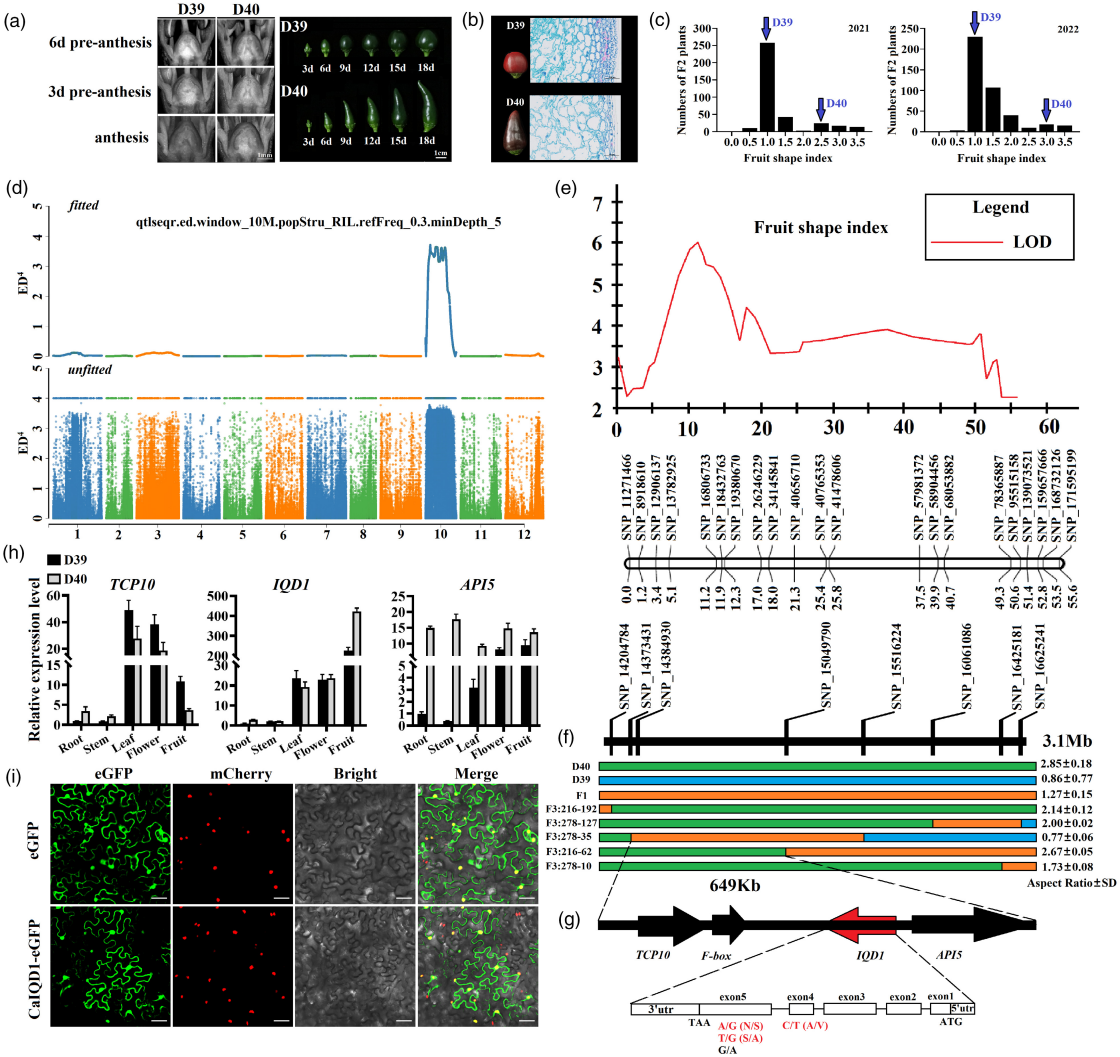
Genetic mapping of *Cafs1* and cloning of *CaIQD1*. (a, b) Fruit phenotypes and cytological observations of the two parents: D39 with round fruit and D40 with long fruit. Scale bars = 1 mm and 1 cm in (a) and 100 μm in (b). (c) Fruit shape index (FSI) in the F_2_ populations of 2021 (left) and 2022 (right). (d) Identification of the fruit shape QTL *Cafs1* by QTL‐seq on the basis of Euclidean distance (ED). (e–g) Fine mapping of the fruit shape QTL *Cafs1*. (h) RT‐qPCR analysis of candidate genes within the target interval. (i) Subcellular localization of CaIQD1 in tobacco leaves. Scale bar = 50 μm.

The fruit of the D39 parent is round, whereas that of D40 is long. Observations of ovary and fruit development through time revealed that the difference in ovary/fruit shape between the two parents occurred mainly after flowering (Figure 1a). Cytological observations revealed that the round fruit of D39 contained many small cells, whereas the long fruit of D40 had fewer but more elongated cells, especially near the outer skin (Figure 1b). The fruit shape of their F1 progeny tended to be intermediate between the parents (Figure S1a). However, fruit shape was diverse among the F_2_ individuals derived from this cross in two consecutive years, and FSI showed a normal distribution (Figure 1c), indicating that the fruit shape trait is quantitative and controlled by multiple genes.

### Identification of a main QTL associated with fruit shape

After sequencing the round fruit and long fruit pools, we identified 2 877 045 SNPs. A graph of Δ(SNP‐index) versus genome position (Figure S1b) revealed a clear peak from 5 to 262 Mb on chromosome 10. Because this initial QTL interval, which we named ‘*Cafs1*’, was very large, covering almost all of chromosome 10, we used the ED method to reconfirm the initial location. The interval was still very large, but the same peak region was visible (Figure 1d), suggesting that it contained a candidate interval that controlled FSI.

### Fine mapping of *Cafs1* and characterization of the *Cafs1* candidate gene

KASP markers (Table S1) were developed from SNPs in the candidate interval. Linkage analysis with Join Map4.0 revealed that the 21 markers were in one linkage group. QTLs were then predicted with MapQTL4.0 using the genetic map and the phenotype data of the F_2_ plants (Tables S2–S4). A QTL associated with fruit shape was identified between SNP_13782925 and SNP_159657666, and the marker with the highest LOD was SNP_16806733 (Table S5). The corresponding physical location between SNP_13782925 and SNP_159657666 spans from 13.4 Mb to 155.9 Mb on chromosome 10, which is within the interval predicted by our bulked segregant analysis. We next screened nine recombinant plants from the F_2_ population using SNP_13782925 and SNP_16806733 and measured FSI in the F_3_ generation. On the basis of these results, eight new KASP markers were developed and used to narrow down the location of *Cafs1* to a 649‐kb interval delimited by SNP_14384930 and SNP_15049790 (Figure 1e–g; Table S6).

Only four genes were annotated within the 649‐kb candidate interval: *TCP10*, an *F‐box*, *Apoptosis inhibitor 5‐like* and *IQ‐DOMAIN 1* (*CaIQD1*) (Figure 1g). Variation information for the interval is provided in Table S7. Gene functional predictions and previous research suggested a potential role for *IQD* family genes in fruit shape regulation. Tissue‐specific expression analysis revealed that *F‐box* was not expressed in either parent, and of the remaining three genes, only *CaIQD1* was highly expressed in the fruit (Figure 1h). Cloning of the *CaIQD1* gene showed that in the *CaIQD1* CDS, there were four SNPs between the parents: one nonsynonymous mutation in the fourth exon and one synonymous mutation and two nonsynonymous mutations in the fifth exon (Figure 1g). We therefore concluded that *CaIQD1* was a candidate gene for *Cafs1*. Phylogenetic analysis of CaIQD1 and 100 homologous proteins from other plant species showed that CaIQD1 was highly conserved in Solanaceae, especially in *Capsicum*, and CaIQD1 was closely related to PHT35739.1 (hypothetical protein from *Capsicum baccatum*), XP_016545680.2 (hypothetical protein from *C. annuum*) and PHU04383.1 (hypothetical protein from *Capsicum chinense*). However, some IQD proteins that have been characterized as regulating fruit shape were not among the top 100 hits, such as SlSUN (NP_001233793.1), SlIQD1 (XM_004234922.4), OsIQD1 (XM_015793411.2), AtIQD2 (NP_568110.2) and others, indicating that CaIQD1 is a new uncharacterized IQD protein (Figure S2a). Transient expression of 35S:CaIQD1‐GFP in *N. benthamiana* leaf cells showed that CaIQD1 was expressed in both the nucleus and the cell membrane (Figure 1i).

### CaIQD1 positively regulates the length–width ratio of pepper fruit

**Figure 2:**
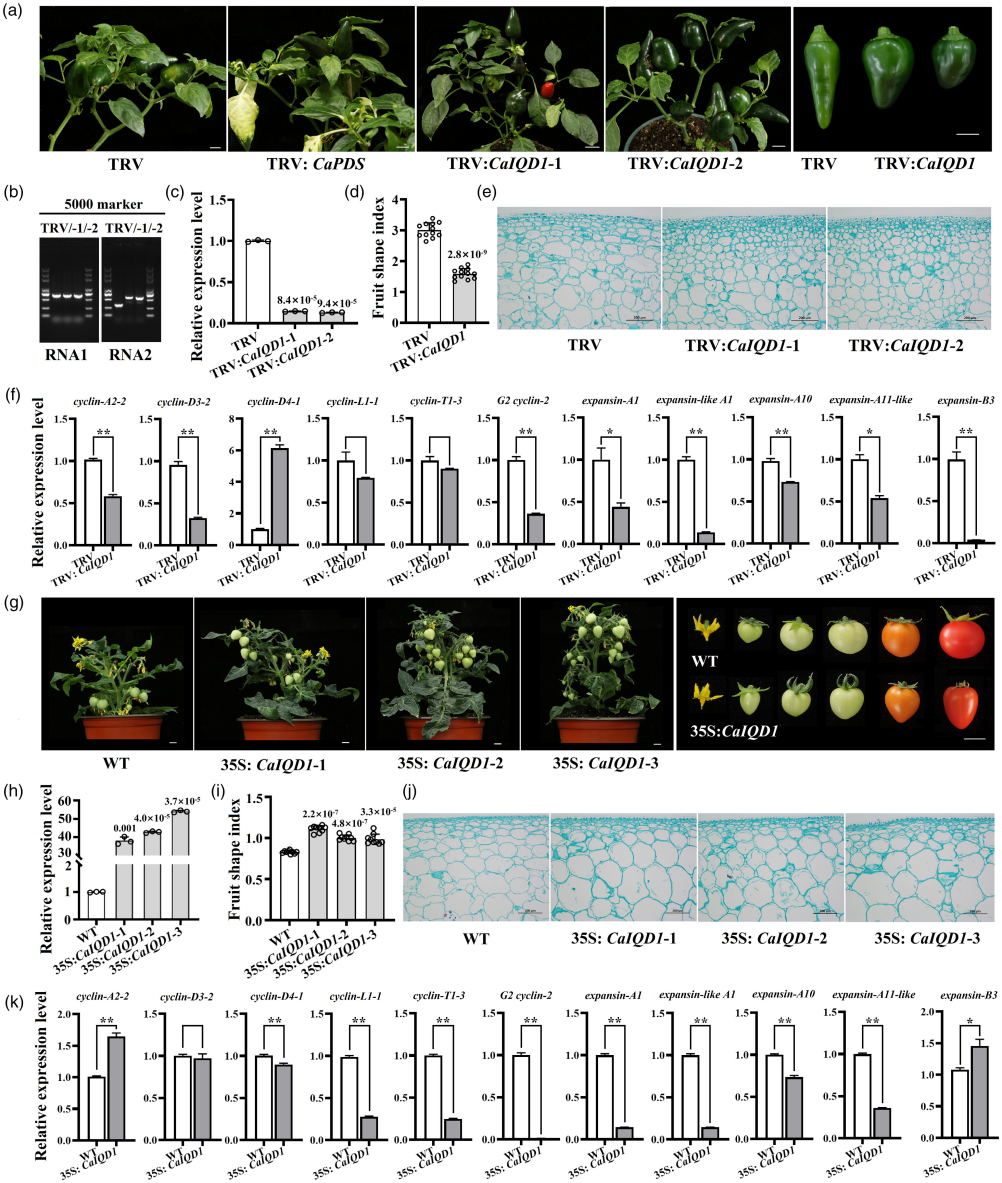
CaIQD1 regulates the shape of pepper and tomato fruit. (a) Fruit morphology of *CaIQD1*‐silenced D40 pepper. TRV and TRV‐*PDS* constructs served as controls. Scale bar = 1 cm. (b, c) Identification and analysis of TRV‐mediated *CaIQD1*‐silenced lines. (d) FSI in TRV:*CaIQD1*. Data were obtained from 12 fruits of four *CaIQD1*‐silenced lines. (e) Cell morphology of representative longitudinal sections of TRV and TRV:*CaIQD1* fruits. Scale bar = 200 μm. (f) Expression of cell cycle and cell expansion‐related genes in TRV:*CaIQD1*. Two‐tailed Student's *t*‐test was used to evaluate the significance of differences. *0.01 < *P* < 0.05. **P < 0.01. (g) Morphology of tomato ‘Micro Tom’ fruits heterologously overexpressing *CaIQD1*. Scale bar = 1 cm. (h) Identification and analysis of *CaIQD1* overexpression lines. (i) FSI in 35S:*CaIQD1*. The data were collected from 27 fruits produced at similar nodes in three *CaIQD1* overexpression lines. (j) Cell morphology of representative longitudinal sections of WT and 35S:*CaIQD1* tomato fruits. Scale bar = 200 μm. Statistical significance was evaluated using two‐tailed Student's *t*‐tests, and exact *P* values are indicated on the graph. (k) Expression of cell cycle and cell expansion‐related genes in 35S:*CaIQD1*. Two‐tailed Student's *t*‐test was used to evaluate the significance of differences. *0.01 < *P* < 0.05. **P < 0.01.

To confirm that *CaIQD1* was indeed the basis of *Cafs1*, we used the VIGS system to silence expression of *CaIQD1* in the long fruited parent D40. Plants expressing the positive control vector TRV:*PDS* showed a clear albino phenotype, confirming that the VIGS procedure was successful (Figure 2a). PCR of peppers inoculated with the empty vector TRV produced 647‐bp (pTRV1) and 320‐bp (pTRV2) target bands, whereas PCR of peppers inoculated with TRV:*CaIQD1* produced 647‐bp (pTRV1) and 564‐bp (pTRV2‐CaIQD1) target bands (Figure 2b), confirming that the relevant recombinant vector is successfully inoculated and expressed. *CaIQD1* expression was significantly lower in pepper fruits infected with TRV:*CaIQD1* than in fruits infected with TRV (Figure 2c), confirming that *CaIQD1* was effectively silenced in TRV:*CaIQD1*‐infected peppers. FSI of the silenced pepper line was reduced by 45% compared with that of the TRV control (Figure 2d), with length reduced by 32% and width increased by 23% (Figure S2b,c). Longitudinal sections of TRV: *CaIQD1*‐1 and TRV: *CaIQD1*‐2 fruits showed that the number of cells increased significantly after CaIQD1 silencing, especially in the exocarp (the outermost three layers of epidermal cells of the fruit); many cells in the fruit peel shortened in length, and their cell morphology changed (Figures 2e and S2d). The expression of many cell cycle and cell expansion genes was significantly affected by reduced CaIQD1 expression (Figure 2f).

Because the efficiency of pepper genetic transformation is low and the transformed plants are difficult to regenerate, we heterologously overexpressed *CaIQD1* in tomato to obtain sufficient numbers of stably transformed plants for fruit phenotyping. We obtained three stable transgenic overexpression lines (Figure 2g), and *CaIQD1* transcript levels were 38.4, 42.8 and 54.1 times higher in the 35S:*CaIQD1*‐1, 35S:*CaIQD1*‐2 and 35S:*CaIQD1*‐3 lines than in the wild type (WT) (Figure 2h). Tomato fruit shape was altered from round in the WT to long and conical in the overexpression lines (Figure 2g), and fruit width changed more significantly than fruit length (Figure S2e,f). Likewise, FSI was significantly higher in the overexpression lines (0.93–1.16) than in the WT (0.8–0.86) (Figure 2i). Longitudinal sections showed no difference in the overall number of cells in the pericarp between the wild type and the 35S: *CaIQD1*‐1, 35S: *CaIQD1*‐2 and 35S: *CaIQD1*‐3 overexpression lines, but the number of cells near the outer pericarp in the overexpression lines increased compared with the control, while the number of cells in the remaining cell layers decreased and became larger. There were differences in the shape and arrangement of cells in the overall pericarp sections between the wild type and the overexpression lines (Figures 2j and S2g). Overexpression of *CaIQD1* also significantly affected the expression of many cell cycle and expansion genes (Figure 2k). Together, these results indicate that the expression of *CaIQD1* can change the fruit shape of pepper and tomato.

### Evidence for interactions among CaOFP20, CaTRM‐like and CaIQD1

**Figure 3:**
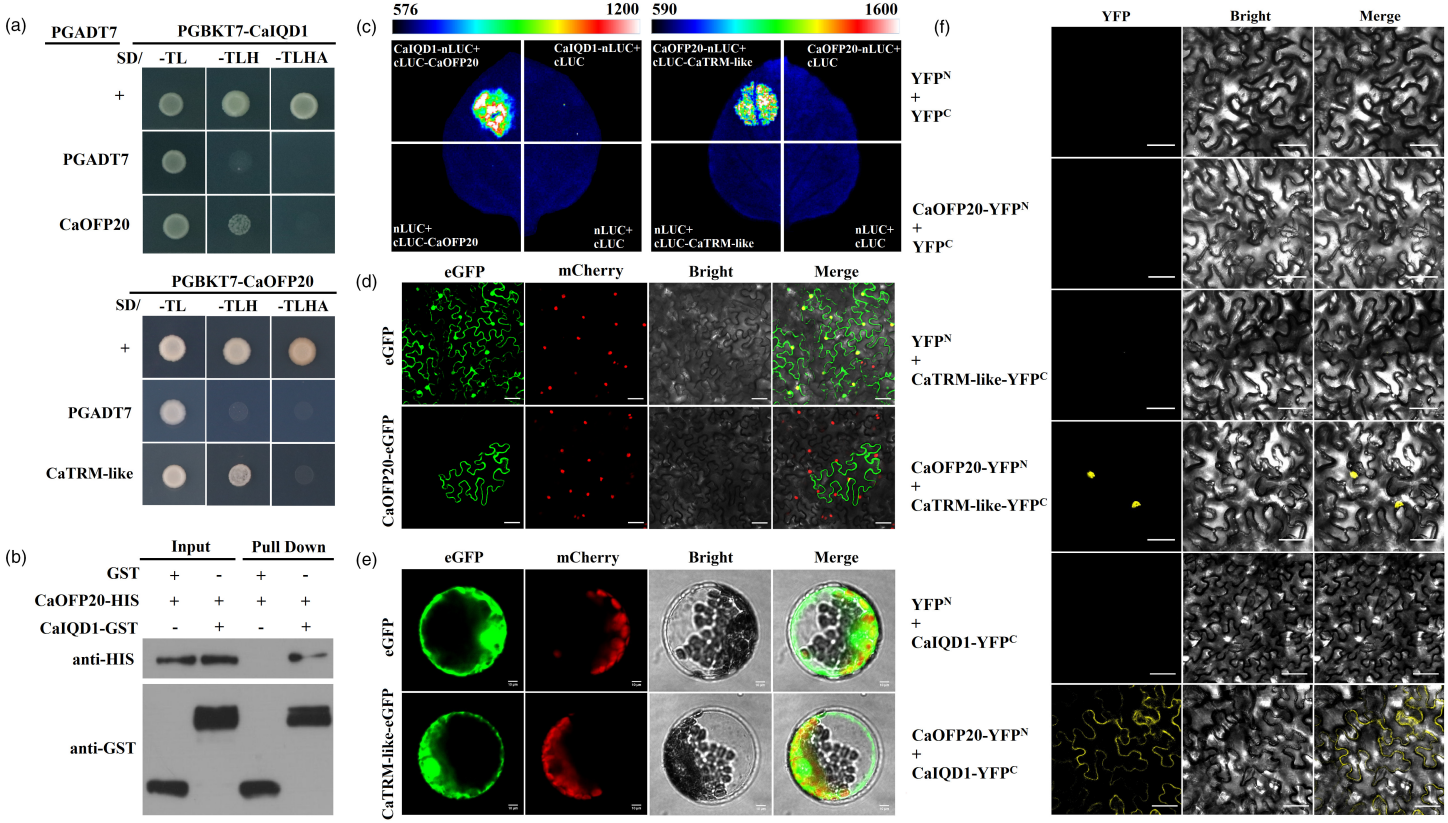
Experimental evidence for interactions among CaOFP20, CaTRM‐like and CaIQD1. (a) Y2H assays. (b) Pull‐down assay confirming the interaction of CaIQD1 and CaOFP20 *in vitro*. (c) Split‐luciferase assay confirming the interactions among CaTRM‐like, CaOFP20 and CaIQD1 *in vivo*. (d) Subcellular localization of CaOFP20 in tobacco leaves. Scale bar = 50 μm. (e) Subcellular localization of CaTRM‐like in tobacco protoplasts. Scale bar = 10 μm. (f) BiFC assay in tobacco leaves demonstrating the interaction of CaIQD1 with CaOFP20 at the cell membrane and of CaTRM‐like with CaOFP20 in the nucleus. Scale bars = 50 μm.

TRM‐OFP and IQD pathways have been repeatedly reported as conserved pathways regulating fruit shape in tomatoes, cucumbers, rice, etc. (Li *et al*., 2023; Snouffer *et al*., 2020; Wu *et al*., 2018; Zhang *et al*., 2023). After confirming that *CaIQD1* positively regulates FSI in pepper fruit, we performed Y2H analysis to identify possible targets in TRM‐OFP that may be involved in the CaIQD1 regulation of the fruit shape pathway and discovered the interaction relationship between CaOFP20, CaTRM‐like and CaIQD1 (Figure S3). Full‐length CaOFP20 and CaIQD1 showed no autoactivation, and yeast cells transformed with the CaOFP20‐BD/CaTRM‐like‐AD and CaIQD1‐BD/CaOFP20‐AD combinations grew well on SD‐Leu‐Trp‐His selective medium (Figure 3a). In an *in vitro* pull‐down experiment, His‐CaOFP20 was pulled down by GST‐CaIQD1, indicating that CaOFP20 directly interacts with CaIQD1 (Figure 3b). Interactions among CaIQD1, CaOFP20 and CaTRM‐like were also verified in split firefly luciferase complementation (SFLC) assays in which a fluorescent signal was detected upon co‐injection of cLUC‐CaTRM‐like with CaOFP20‐nLUC and cLUC‐CaOFP20 with CaIQD1‐nLUC (Figure 3c). Subcellular localization assays in tobacco leaves (CaOFP20) and protoplasts (CaTRM‐like) indicated that CaOFP20 was expressed in the cell membrane (Figure 3d), and CaTRM‐like in the cytoplasm and nucleus (Figure 3e). However, bimolecular fluorescence complementation (BiFC) assays indicated that the CaOFP20–CaIQD1 have interaction and may occur at the cell membrane, whereas the CaTRM‐like–CaOFP20 have interaction and may occur in the nucleus (Figure 3f).

### Both CaTRM‐like and CaOFP20 influence pepper fruit shape

**Figure 4:**
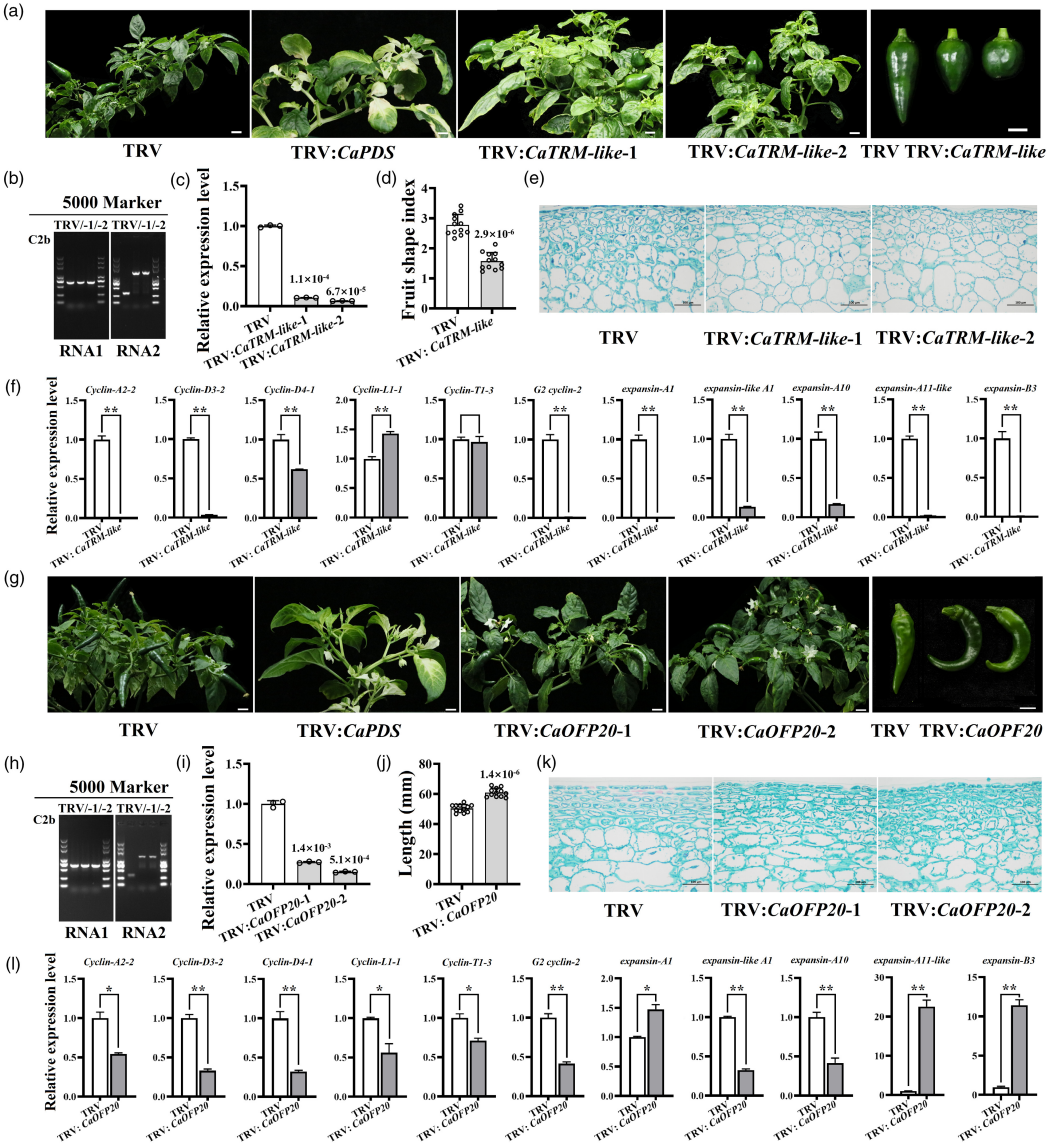
CaTRM‐like and CaOFP20 regulate pepper fruit shape. (a) Fruit morphology of D40 pepper upon *CaTRM‐like* silencing. Scale bar = 1 cm. (b, c) Identification and analysis of TRV‐mediated *CaTRM*‐like‐silenced lines. (d) FSI of TRV:*CaTRM‐like* plants. Data were obtained from 12 fruits of four *CaTRM‐like*‐silenced lines. (e) Cell morphology in representative longitudinal sections of TRV and TRV:*CaTRM‐like* fruits. Scale bar = 100 μm. (f) Expression of cell cycle and cell expansion‐related genes in TRV:*CaTRM‐like*. Two‐tailed Student's *t*‐test was used to evaluate the significance of differences. *0.01 < *P* < 0.05. **P < 0.01. (g) Fruit morphology of Zunla pepper upon *CaOFP20* silencing. Scale bar = 1 cm. (h, i) Identification and analysis of TRV‐mediated *CaOFP20*‐silenced lines. (j) Fruit length of TRV:*CaOFP20*. Data were obtained from 12 fruits of four *CaOFP20*‐silenced lines. (k) Cell morphology in representative longitudinal sections of TRV and TRV:*CaOFP20* fruits. Scale bar = 100 μm. The significance of differences was evaluated using two‐tailed Student's *t*‐tests, and exact *P* values are indicated on the graph. (l) Expression of cell cycle and cell expansion‐related genes in TRV:*CaOFP20*. Two‐tailed Student's *t*‐test was used to evaluate the significance of differences. *0.01 < *P* < 0.05. **P < 0.01.

To determine whether CaOFP20 and CaTRM‐like also regulate pepper fruit shape, we silenced their expression using the highly efficient pepper flower‐ and fruit‐specific silencing vector pTRV2‐C2b based on TRV vector transformation (Figure 4a). Target bands of 647 bp (pTRV1), 971 bp (PTRV2‐C2b) and 1388 bp (pTRV‐C2b‐*CaTRM‐like*) were amplified from the *CaTRM‐like*‐silenced plants, confirming that the relevant recombinant vector is successfully inoculated and expressed (Figure 4b). Expression of *CaTRM‐like* was significantly reduced in pepper fruits infected with TRV:*CaTRM‐like* (Figure 4c), and the FSI of the silenced plants was reduced by 43% relative to that of the TRV controls (Figure 4d). Specifically, fruit length decreased by 29%, and fruit width increased by 19% (Figure S4a,b). Longitudinal sections showed that although the cells in the outermost fruit peel cells became more numerous and smaller like TRV: *CaIQD1*, the overall number of cells in *CaTRM‐like*‐silenced fruits decreased and the cell size increased compared with the TRV control (Figures 4e and S4c). The expression of many cell cycle and expansion genes was significantly affected by reduced *CaTRM‐like* expression (Figure 4f). Flowers of the *CaTRM‐like*‐silenced lines also showed significant changes, including wrinkled and smaller petals and significantly shorter stigmas. Further analysis of the expression of *CaTRM*‐like in different tissues revealed that it was also highly expressed in flowers (Figure S4d–h), suggesting that CaTRM‐like regulates the development of pepper flowers.

Similarly, a 1274‐bp (PTRV2‐C2b‐*CaOFP20*) band was amplified from peppers infected with TRV:*CaOFP20*. *CaOFP20* expression was significantly reduced in the fruits of these silenced plants compared with TRV plants, fruit length was 20% higher, and the fruits exhibited a distinctive curled phenotype (Figure 4g–j). Longitudinal sections showed that the number of cells in the *CaOFP20*‐silenced lines increased significantly, and some cells in the mesocarp and endocarp were significantly elongated. In TRV fruits, the number of cells near the outer epidermis increased and was arranged neatly, while the cells near the outer epidermis of the *CaOFP20*‐silenced lines were disorganized (Figures 4k and S4i). The expression of many cell cycle and expansion genes was also significantly affected by reduced *CaOFP20* expression (Figure 4l). Thus, CaOFP20 appears to negatively regulate fruit length and curvature in pepper.

### CaIQD1, CaOFP20 and CaTRM‐like share some similar effects on pepper fruit shape

We found that reductions in *CaIQD1* and *CaTRM‐like* expression caused reductions in pepper FSI and that reduced expression of *CaOFP20* caused increases in pepper fruit length and curvature. Cytological observations suggested that CaIQD1 affected pepper fruit shape by influencing cell proliferation, arrangement, shape and size, and that CaOFP20 and CaTRM‐like affected fruit shape by regulating cell division and cell arrangement (Figures 2 and 4); the three proteins thus showed some similarities in their effects.

Interestingly, although silencing *CaIQD1* and *CaTRM‐like* produced rounder fruits, whereas silencing *CaOFP20* produced longer, curled fruits, the silencing of the three genes downregulated the expression of most of these cyclin and expansion genes (Figures 2f,k and 4f,l), and the number of cells in the outer epidermis layers of these silenced lines was also increased. Besides, the effects of *CaIQD1* and *CaTRM‐like* silencing were most similar: the expression of all tested expansin genes was downregulated in the fruit, as was the expression of *cyclin A2‐2*, *cyclin D3‐2* and *G2 cyclin‐2*. The effects of *CaOFP20* silencing were broadly similar, but in this case, all tested cyclin genes were downregulated, and three expansin genes were upregulated. 35:*CaIQD1* also has many similarities in the effects on the expression of cell cycle and expansion genes in *CaIQD1* transgenic tomato fruits and *CaTRM‐like*‐silenced fruits; however, given the differences between species, this comparison must be interpreted cautiously. In addition, in the functional validation plants of these three genes, the expression changes of *cyclin‐D4‐1*, *expansin‐A1* and *expansin‐A11‐like* showed different trends compared with other genes, especially *cyclin‐D4‐1*, which may have various functions. Overall, silencing genes with opposite effects on fruit shape had many similar effects on cyclin and expansin expression, downregulating the expression of most of these cell cycle and expansion‐related genes. This result may reflect some compensatory mechanism between cell proliferation and cell expansion. In addition, the tissue‐ and cell‐type‐specific effects of *CaIQD1*, *CaTRM‐like* and *CaOFP20* on the expression of cyclin and expansin genes may also be masked by the sampling of a large number of fruit tissues. Nonetheless, these results underscore the central regulatory role played by CaIQD1 and its interacting partners in the basic processes controlling cell division and growth.

Additional Y2H and LCI experiments showed that CaOFP20 also interacts with the IQD family members SUN, IQD3 and IQD17 (Figure S5a,b), which are on different evolutionary branches of the phylogenetic tree from CaIQD1 (Figure S5c). This suggests that CaIQD1, CaOFP20 and CaTRM‐like may play a role in regulating fruit shape in the same genetic pathway and that regulatory interactions between CaOFP20 and multiple IQD family members may be somewhat conserved.

### Regulation of fruit shape by CaIQD1 may involve interactions with MTs and Ca^2+^‐CAM

**Figure 5:**
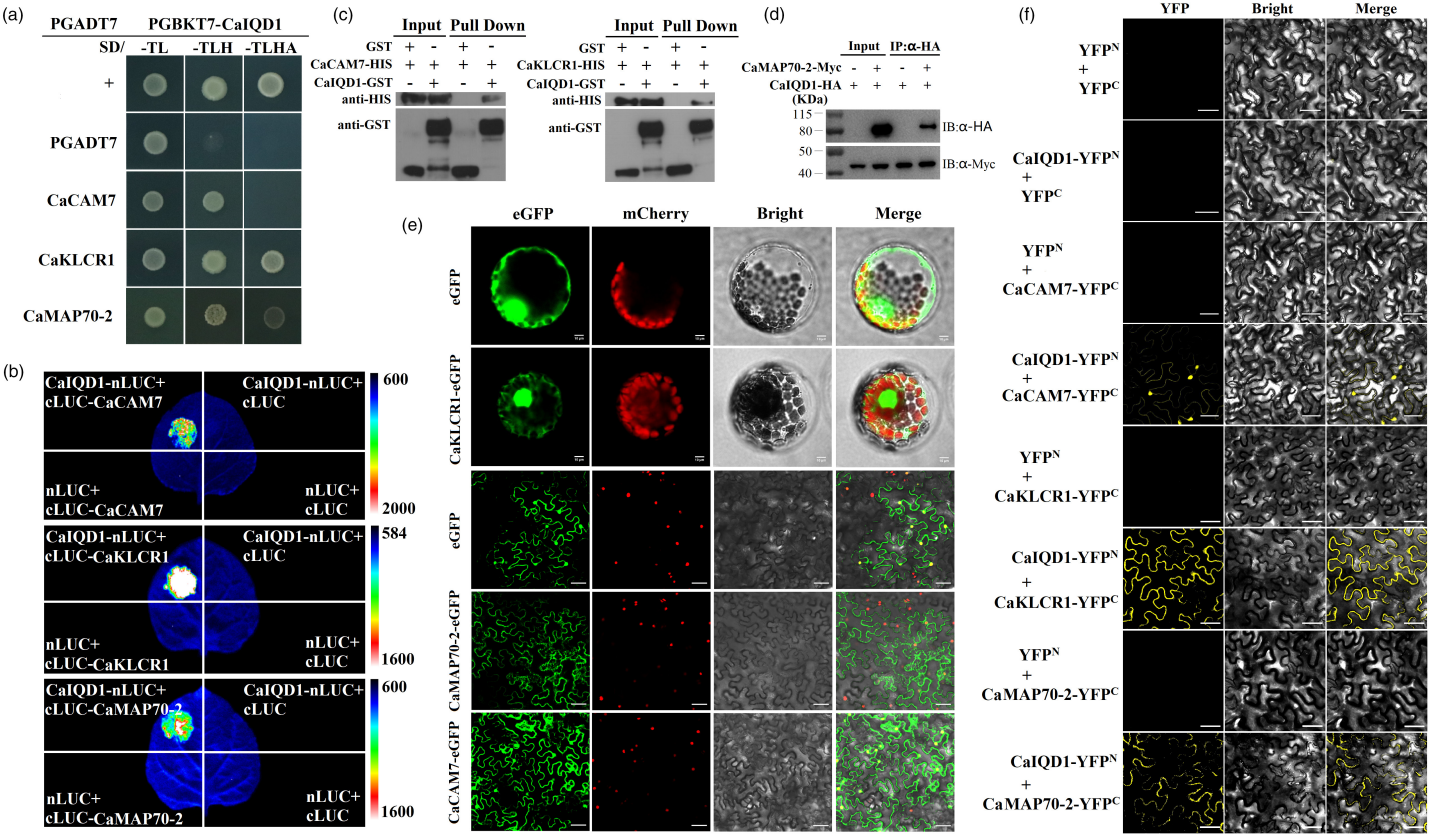
Experimental evidence for interactions of CaIQD1 with CaKLCR1, CaMAP70‐2 and CaCAM7. (a) Y2H assays were used to detect interactions of CaIQD1 with CaKLCR1, CaMAP70‐2 and CaCAM7. (b) Interactions between CaIQD1 and CaKLCR1, CaMAP70‐2 and CaCAM7 were verified *in vivo* by split‐luciferase assays. (c) Interactions of CaIQD1 with CaKLCR1 and CaCAM7 *in vitro* were confirmed by pull‐down assays. (d) A co‐immunoprecipitation assay confirmed the interaction between CaIQD1 and CaMAP70‐2. (e) Localization of CaKLCR1 in tobacco protoplasts (scale bar = 10 μm) and of CaMAP70‐2 and CaCAM7 in tobacco leaves (scale bar = 50 μm). (f) BiFC assay demonstrating the interactions of CaIQD1 with CaKLCR1 and CaMAP70‐2 at the cell membrane in tobacco leaves and of CaIQD1 with CaCAM7 at the cell membrane and in the nucleus in tobacco leaves. Scale bar = 50 μm.

In addition to causing changes in cell number, we noticed that the regulation of pepper fruit shape by *CaIQD1* also caused significant changes in cell morphology (Figure 2e, j). Cytoskeleton‐related proteins have been reported to participate in fruit shape development through the arrangement and reorganization of microtubules, which can significantly cause changes in cell morphology (Bao *et al*., 2023, 2024; Duan *et al*., 2017; Zang *et al*., 2021). So, we explored potential interactions between cytoskeleton‐related proteins and CaIQD1. Y2H experiments revealed direct interactions of CaIQD1 with the MT motor protein CaKLCR1 and the MT‐binding protein CaMAP70‐2 (Figure 5a). Yeast cells expressing the CaIQD1‐BD/CaKLCR1‐AD and CaIQD1‐BD/CaMAP70‐2‐AD vector combinations grew well on selective SD‐Leu‐Trp‐His and SD‐Leu‐Trp‐His‐Ade media, respectively. Notably, CaIQD1 also interacted directly with the calmodulin CaCAM7 (Figure 5a), and Ca^2+^‐CAM has been reported to directly mediate microtubule rearrangement and regulate cytoskeletal dynamics (Bürstenbinder *et al*., 2013, 2017). The interactions among CaCAM7, CaIQD1 and CaKLCR1/CaMAP70‐2 were also verified by SFLC assays. Fluorescence signals were detected at the co‐injection sites of CaIQD1‐nLUC + cLUC‐CaCAM7, CaIQD1‐nLUC + cLUC‐CaKLCR1 and nLUC‐CaIQD1 + cLUC‐CaMAP70‐2 (Figure 5b). Likewise, His‐CaCAM7 and His‐CaKLCR1 were all pulled down by GST‐CaIQD1 in pull‐down assays (Figure 5c), confirming that CaCAM7 and CaKLCR1 interact directly with CaIQD1 *in vitro*. The specific interaction between CaIQD1 and CaMAP70‐2 was also further verified by co‐immunoprecipitation (Co‐IP) analysis (Figure 5d).

Subcellular localization assays showed that CaCAM7 was expressed in the nucleus and at the cell membrane (Figure 5e), KLCR1 in the nucleus and cytoplasm, and CaMAP70‐2 at the cell membrane and in the cytoplasm (Figure 5e). However, BiFC assays revealed that the CaIQD1–CaKLCR1 and CaIQD1–CaMAP70‐2 have interactions and may occur at the cell membrane, and the CaCAM7–CaIQD1 have interactions and may occur at the cell membrane and in the nucleus (Figure 5f). We also found that changes in CaIQD1 expression significantly affected the expression of microtubule‐associated genes *MAP70‐1*, *MAP70‐2*, *KCLR1*, *KCLR3*, *TUBB1*, *TUBB2*, *KIN4* and *KIN7* (Figure S6). These results suggest that CaIQD1 serves to physically connect Ca^2+^‐CAM with cytoskeletal proteins and that the regulation of fruit shape by CaIQD1 may be related to changes in cell shape controlled by MTs.

### Protein interactions between CaIQD1 and CaDOF1.4/CaIQD3/CaSUN/CaIQD17

**Figure 6:**
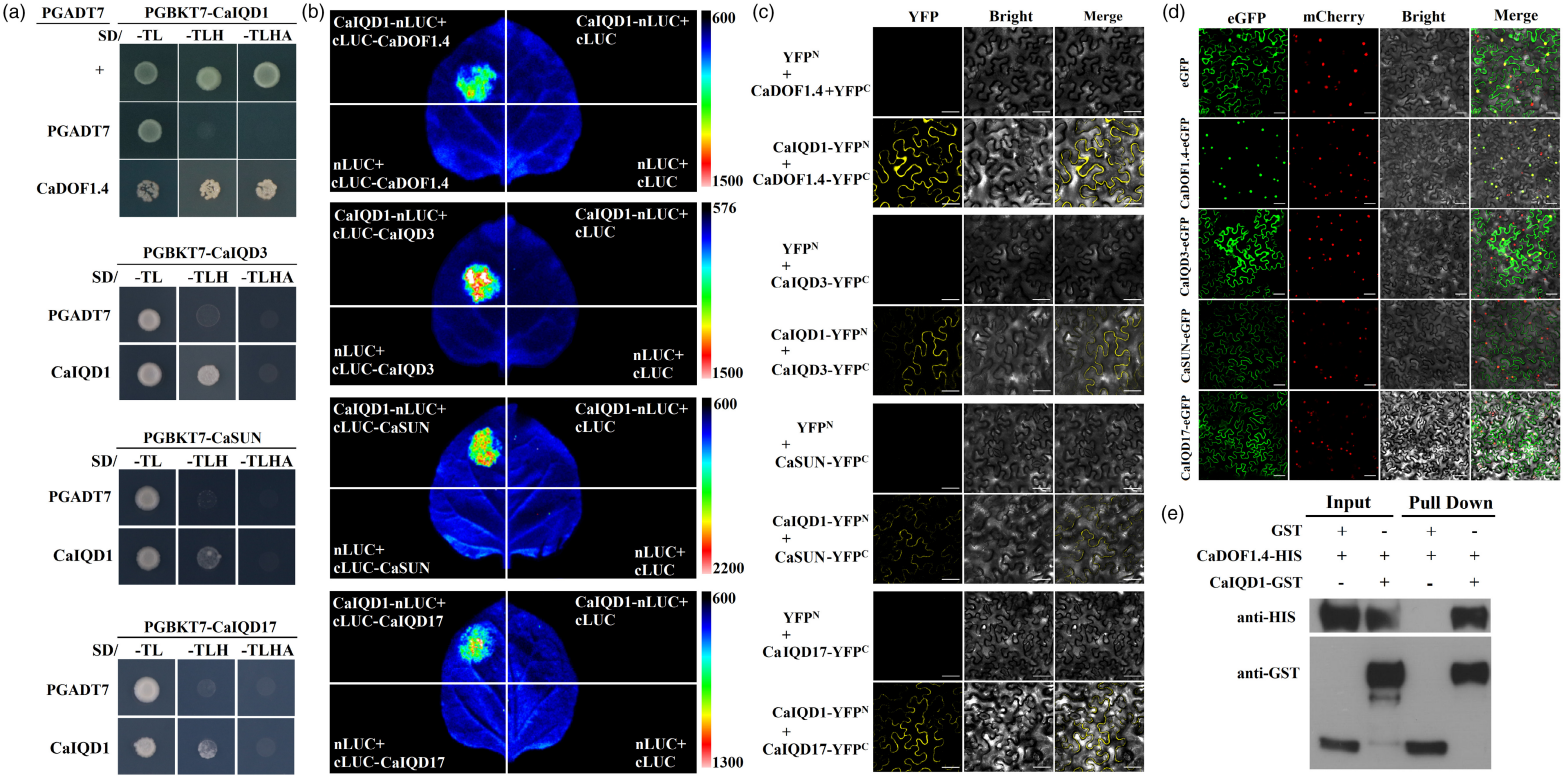
Experimental evidence for the interactions between CaIQD1 and CaDOF1.4/CaIQD3/CaSUN/CaIQD17. (a) Y2H assays revealed interactions between CaIQD1 and CaDOF1.4/CaIQD3/CaSUN/CaIQD17. (b) Interactions between CaIQD1 and CaDOF1.4/CaIQD3/CaSUN/CaIQD17 were verified *in vivo* by split‐luciferase assays. (c) BiFC assays demonstrated that the interactions between CaIQD1 and CaDOF1.4/CaIQD3/CaSUN/CaIQD17 all occurred at the cell membrane in tobacco leaves. Scale bar = 50 μm. (d) Subcellular localization of CaDOF1.4, CaIQD3, CaSUN and CaIQD17 in tobacco leaves. Scale bar = 50 μm. (e) The interaction between CaIQD1 and CaDOF1.4 was confirmed *in vitro* by a pull‐down assay.

To further characterize the interaction network of CaIQD1 in the regulation of fruit shape, we explored the relationship between CaIQD1 and homologues of several typical genes that have been shown to regulate fruit shape in other species (Kim *et al*., 2021; Xiao *et al*., 2008; Yang *et al*., 2020; Zang *et al*., 2021). Y2H and LCI assays revealed that CaIQD1 interacted with CaDOF1.4, CaIQD3, CaSUN and CaIQD17 (Figure 6a,b), and BiFC assays showed that there was an interaction between them and that they might all occur at the cell membrane (Figure 6c). Subcellular localization results showed that CaDOF1.4 itself was located in the nucleus, CaIQD3 was expressed in the nucleus and at the cell membrane, and CaSUN and CaIQD17 were expressed at the cell membrane (Figure 6d). GST pull‐down assays also showed a direct interaction between CaIQD1 and CaDOF1.4 (Figure 6e). These results indicate that CaDOF1.4, CaIQD3, CaSUN and CaIQD17 interact with CaIQD1 and may also form part of the network that regulates fruit shape in pepper.

## Discussion

### Interactions among IQD, OFP and TRM regulate cell expansion and division to control fruit shape and are potentially conserved

Previous work in other plant species has provided insights into the potential mechanisms of IQD, OFP and TRM family proteins in influencing fruit shape. For example, the IQD family *SUN* changes tomato fruit shape by affecting longitudinal/transverse divisions of fruit cells (Xiao *et al*., 2008); *OVATE* and *SlOFP20* in tomatoes regulate fruit shape by inhibiting longitudinal but promoting transverse cell divisions (Boualem *et al*., 2022; Snouffer *et al*., 2020); and *TRM* is an important regulator of rice grain shape, Arabidopsis silique shape and cucumber and tomato fruit shape, regulating fruit shape by affecting cell division direction and cell expansion (Lee *et al*., 2006; Zhang *et al*., 2023; Zhu *et al*., 2023; Xie *et al*., 2023). Snouffer *et al*. (2020) also proposed that OFP controls the localization of TRM on MTs and that TRM–OFP interactions affect MT organization to control shape elongation; mutants with reduced or no expression of OFP exhibit organ elongation and TRM localization defects, while mutants with reduced or no expression of TRM exhibit organ flattening, and these shape changes may also be affected by other shape regulators (such as IQD). Despite this, the interaction relationship and mechanism of action between IQD and TRM‐OFP remain unclear.

In this study, we cloned an IQD protein (CaIQD1) that regulates the shape of the pepper fruit and clarified its protein interaction relationship with TRM‐OFP. That is, CaIQD1 directly interacts with CaOFP20 but does not interact with CaTRM‐like. The three proteins did not form a complex to regulate the shape of the fruit. The effect of CaTRM‐like/CaOFP20/CaIQD1 on the shape of the fruit was accompanied by cell division, expansion and morphology. We also observed some interesting phenomena, such as silencing *CaIQD1*, CaTRM‐like and *CaOFP20*, downregulated the expression of many cell cycle and expansion‐related genes, and that the fruit phenotypes of overexpressed and TRV lines differed but the number of cells near the outer epidermis seemed to increase. Anticlinal cell division, which is most common in tomato peel and is the dominant pattern in fleshy fruits, increases the number of cells in a certain layer. The outermost pericarp cell (E1) changes the number of cells through anticlinal division, thus driving changes in fruit volume and circumference (Mauxion *et al*., 2021; Renaudin *et al*., 2017). This suggests that the regulation of fruit morphogenesis by CaIQD1, CaTRM‐like and CaOFP20 may be carried out through the same pathway related to cell division and expansion. In addition, although *CaIQD1* and *CaTRM‐like* silencing lead to similar fruit phenotypes, there are obvious differences in the overall changes in their fruit pericarp cells; in tomatoes, it was found that the gain and loss of function of *SlCCS52A* (cell cycle switch gene) lead to similar fruit phenotypes, but there are differences at the overall cell level (Mathieu‐Rivet *et al*., 2010). This suggests some differences in the regulatory mechanisms of fruit morphology behind *CaIQD1* and *CaTRM‐like*, especially in affecting the mode of cell division. For example, in this manuscript, their effects on the expression of cell cycle genes *cyclin‐D4‐1* and *cyclin‐L1‐1* are inconsistent.

In summary, consider that changes in fruit shape involve the influence of a series of overlapping and interrelated cellular events such as the direction, duration, rate of cell division and isotropic and anisotropic cell expansion, which can occur alone or in combination, occurring in different regions of the fruit (Renaudin *et al*., 2017; van der Knaap and Østergaard, 2017; Mauxion *et al*., 2021). These specific changes are related to the exact action and mechanism of each fruit shape regulator. Therefore, based on identifying the interaction between CaIQD1, CaTRM‐like and CaOFP20 and understanding their regulatory effects on fruit shape, more evidence and reasonable combinations of gene mutants (such as changes in cell division and cell expansion) are needed in the future to explore the molecular mechanisms behind them to establish a reliable regulatory network.

### Regulation of fruit shape by CaIQD1 may rely on Ca^2+^‐CAM7‐mediated MT rearrangement

Ca^2+^ signal transduction and dynamic cytoskeletal reorganization are also important for the coordination and control of plant cell shape and growth (Bürstenbinder *et al*., 2017; Levy *et al*., 2005), and IQD proteins have been implicated in both of these processes. Research in Arabidopsis has shown that CaIQD1 interacts with the microtubule‐binding protein CaKLCR1, binds to CaM/CMLs and localizes to the microtubules, suggesting that it may recruit cargo to kinesin motors for microtubule transport (Bürstenbinder *et al*., 2013). Recent work showed that overexpression of IQD proteins altered MT organization and cell shape in Arabidopsis and provided evidence that IQD proteins act as scaffolds, sequestering Ca^2+^‐CaM at specific sites to enable precise control of Ca^2+^‐dependent processes (Bürstenbinder *et al*., 2017). Similarly, the NET3C‐KLCR1‐IQD2 module was shown to act as a bridge between actin and microtubules, thereby affecting plant morphogenesis in Arabidopsis (Zang *et al*., 2021). In this study, the homologous genes of Arabidopsis IQ2 and KLCR1 were identified as CaIQD3 and CaKLCR1 in pepper, respectively. Both CaIQD3 and CaIQD1 exhibited protein interactions with CaCAM7/KLCR1. Thus, CaIQD1 may also act as a scaffold protein in interactions among actin, microtubules and CAM in pepper, although additional work will be required to demonstrate such a mechanism.

Connections among IQD proteins, Ca^2+^‐CAM signalling and microtubule dynamics have also been reported in rice. CAM has been shown to bind to OsIQD14 in a Ca^2+^‐dependent manner, and OsIQD14 regulates microtubule dynamics to regulate grain shape in rice (Yang *et al*., 2020). Similarly, the plasma membrane‐associated IQD protein GSE5, which also regulates rice grain size, interacts with the rice calmodulin OsCaM1‐1 (Duan *et al*., 2017). The pepper ortholog of *OsIQD14* is *CaIQD17*. Here, we showed that CaIQD17 interacts with CaIQD1 and that both CaIQD17 and CaIQD1 interact with CaCAM7. Therefore, a Ca^2+^‐CaCAM7‐CaIQD1 module may influence pepper shape by regulating microtubule dynamics, consistent with the observed changes in fruit cell morphology upon overexpression and silencing of *CaIQD1*.

In tomato, the SIMAP70‐SlIQD21a/SUN10 module regulates fruit morphology through its effects on microtubule dynamics (Bao *et al*., 2023), and the SlMAP65‐1‐IQD26/SUN18 interaction affects fruit morphology through its effects on cell division (Bao *et al*., 2024). The pepper ortholog of SIMAP70 is CaMAP70‐2, another CaIQD1 interaction partner identified in the present study. Although the IQD proteins SlIQD21a and CaIQD1 are not strictly orthologous, the CaMAP70‐2‐CaIQD1 interaction provides additional evidence that CaIQD1 may regulate pepper morphology by affecting microtubule dynamics. At the same time, alterations in *CaIQD1* expression had similar effects on the expression of genes that influence microtubule stability (*MAP70*); mediate microtubule transport, rearrangement and signalling (*KLCR/KIN*) and influence microtubule transport and number (*TUBB*) in TRV: *CaIQD1* and 35S: *CaIQD1*. In addition to *KLCR1*, the detected genes showed a positive regulatory trend between *CaIQD1*. Thus, our results suggest that CaIQD1 acts as a core network hub, interacting directly with the Ca^2+^‐signal‐decoding protein CaCAM7, the MT‐associated protein CaMAP70‐2 and the MT motor protein CaKLCR1 to affect cell morphology.

### Different subcellular localizations of IQD family members may influence their mechanisms of action

To date, there have been a number of reports on how the specific subcellular localizations of IQD family members affect plant morphology. For example, the homologous rice IQD proteins GW5 and GW5L are located at the cell membrane. These proteins influence seed width and weight by regulating BR signalling (Liu *et al*., 2017). OsIQD14 is associated with the periplasmic MTs and affects rice grain shape by regulating the morphology of rice chaff cells (Yang *et al*., 2020). SlIQD21 is also associated with MTs in tomato and regulates tomato fruit morphology through its effects on MT cytoskeletal dynamics (Bao *et al*., 2023). GSE5 influences rice grain shape by affecting cell number, but its subcellular localization has not been reported (Duan *et al*., 2017). In a study that comprehensively characterized the entire Arabidopsis IQD family, Bürstenbinder *et al* found that patterns of IQD localization coincided with patterns of IQD‐dependent CaM recruitment, suggesting that different IQDs regulate Ca^2+^‐dependent processes by recruiting Ca^2+^‐CaM signalling modules to specific sites (Bürstenbinder *et al*., 2017). We therefore speculated that the mechanisms by which IQD family members regulate fruit shape may be related to their different subcellular localizations, a hypothesis that receives some preliminary support in the present work. In this study, CaIQD1 and CaIQD3 were expressed in the nucleus and at the cell membrane (Figures 1i and 6d), while CaIQD17 and CaSUN (located on the same branch of the phylogenetic tree) were only expressed at the cell membrane (Figures 6d and S5c). Y2H assays showed that all four proteins could interact with calmodulin CaCAM7 (Figure S7a–c). CaIQD3, which has the same subcellular localization as CaIQD1, also interacted with CaDOF1.4 and CaKLCR1, whereas CaIQD17 and CaSUN did not (Figure S7a–c). Although two common conserved motifs were present in CaIQD1, CaIQD3, CaSUN and CaIQD17, the motif composition of CaIQD1 was more similar to that of CaIQD3 (Figure S7d). In addition, the subcellular localization position of CaIQD1 was not affected when CaIQD1 and CaKLCR1 were co‐located, but the subcellular localization positions of CaSUN and CaIQD17 were changed when CaSUN and CaIQD17 were co‐located with CaKLCR1, respectively (Figure S8). Therefore, the mechanism of IQD family proteins is full of diversity and complexity. It will be interesting to explore the mechanism of different IQD family proteins, especially whether their different subcellular localizations are part of the mechanistic framework by which they influence plant cell function, shape and growth.

**Figure 7:**
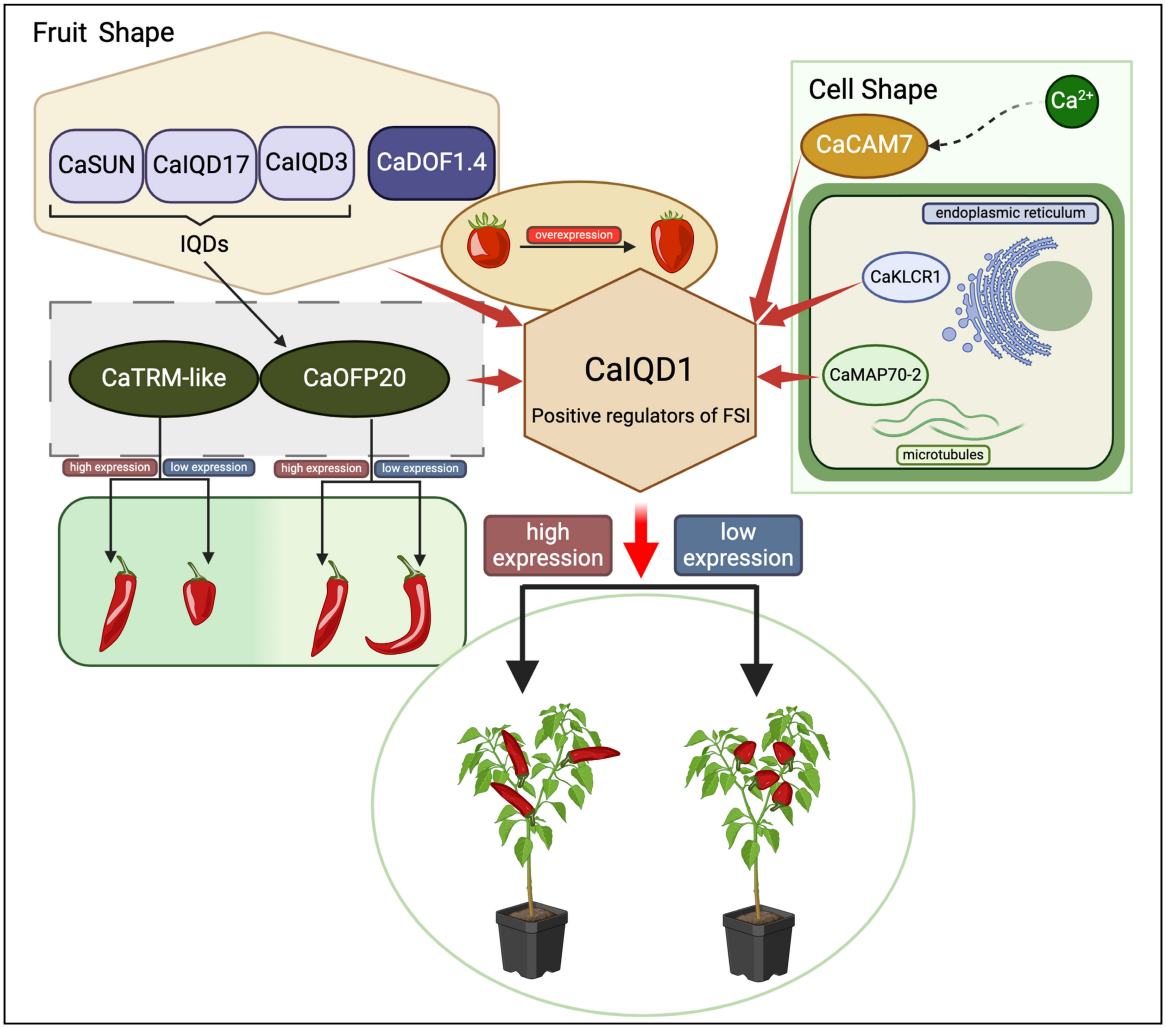
A possible network model of CaIQD1 regulating pepper fruit shape.

In summary, we performed traditional genetic mapping and cloned the gene *CaIQD1 that* controls fruit shape in pepper. Silencing of *CaIQD1* made pepper fruits shorter and wider, whereas its overexpression made tomato fruits more slender. CaIQD1 was shown to interact directly with other known fruit shape regulators. In particular, CaIQD1, CaOFP20 and CaTRM‐like have similar effects on pepper fruit shape and are likely conserved. Nonetheless, the specific mechanisms by which CaIQD1 exerts its effects on fruit shape are complex, involving changes in cell division, number, size and arrangement, as well as interactions with Ca^2+^‐CaM and microtubules. CaIQD1 is likely to act as a core scaffold protein, transducing Ca^2+^ signals to alter the organization and dynamics of the cytoskeleton (Figure 7).

## Materials and methods

### Plant materials and cultivation

We performed a cross using the F_7_‐generation homozygous inbred lines ‘D40’ (with a long fruit phenotype) and ‘D39’ (with a round fruit phenotype) as parents. The resulting F_1_ individuals were selfed to produce an F_2_ segregating population. In January 2021, a small F_2_ population composed of 369 individuals was used for genetic analysis and preliminary mapping of pepper fruit shape genes. Phenotyping was performed by measuring the length‐to‐width ratio (FSI) of three fruits from the same node on each pepper plant. In January 2022, 441 F_2_ populations were obtained after planting, and in July 2022, an F_2:3_ segregating population of 1861 individuals was screened to obtain more recombinants and narrow the positioning interval. All plants were grown at the experimental base of Hunan Agricultural University.

### QTL analysis with next‐generation sequencing data

Based on the FSI phenotyping, we selected 20 extreme individuals with long conical fruits and 20 extreme individuals with round fruits in the F_2_ population for bulked segregant DNA sequencing. The cetyltrimethylammonium bromide (CTAB) method was used to extract DNA from individual samples (Yu *et al*., 2019), and equal amounts of DNA from long or round individuals were combined to obtain two mixed pools for sequencing. Whole‐genome sequencing of the parents and the two mixed pools was performed on the Illumina NovaSeq 6000 platform in 150 PE mode. BWA was used to map clean sequencing reads to the Zunla2 reference genome (Li and Durbin, 2009), and SAMtools was used for SNP calling. Because the parents were high‐generation inbred lines, only homozygous SNPs between the parents were retained; additional SNP thresholds included a base quality value ≥30, a mapping quality value ≥30 and a base depth between 2 and 80 in the two F_2_ pools. The Euclidean distance (ED) and SNP‐index algorithms were used to identify SNPs associated with fruit shape. ED was calculated from the frequencies of the four bases (A,C, T and G) in the long fruit and round fruit mixed pools as described in Hill *et al*. (2013), and a threshold of 1% ED was used for SNP identification on each chromosome. SNP‐index was calculated as the proportion of reads that contained a given SNP at an SNP site relative to the total number of reads mapped to that site (Takagi *et al*., 2013). Δ(SNP‐index) was then defined as the SNP‐index of the long fruit pool minus the SNP‐index of the round fruit pool, and the average Δ(SNP‐index) was calculated in 1‐Mb sliding windows with a 100‐kb step size.

### Fine mapping of a major fruit shape QTL

To confirm and further narrow the candidate QTL interval, we designed Kompetitive allele‐specific PCR (KASP) markers based on SNPs that differed within the interval (http://www.snpway.com/) and used them to genotype the entire F_2_ population of 810 lines. The screening criteria for SNP were MAF ≥0.05 and parental homozygous difference. After the typing results were obtained, phenotypic data (FSI) were combined for linkage analysis. JoinMap4.0 was used to perform linkage analysis of the marker sites (Van Ooijen, 2011), and the Kosambi map function was used to convert the recombination rate into a genetic map distance in order to draw a traditional genetic map. MapQTL4.0 was used with the FSI data to predict possible QTLs using the multi‐QTL mapping method (Lu *et al*., 2014). The scanning step was set to 1.0 cm, and the LOD threshold was set to LOD >3.0. The location of the maximum LOD value was found by drawing the map, that is, the region of the primary QTL locus. Individual recombinant F_2_ plants were screened using markers near the highest point of the QTL, then self‐crossed and grown to seed. Phenotype data were collected from the resulting F_2:3_ population, and additional KASP markers were developed to further narrow the QTL interval.

### Cloning and analysis of candidate genes

Genes located within the candidate interval were identified from the reference genome, and genic mutations in the interval were identified from the resequencing data, paying special attention to mutations that caused changes in amino acid sequences or promoter regions and to genes whose functional annotations were potentially related to fruit shape. Expression of all candidate genes was examined in different tissues of both parents to obtain further evidence for their potential association with fruit shape traits (see Section ‘Evidence for interactions among CaOFP20, CaTRM‐like, and CaIQD1’). Selected candidate genes were cloned, and their protein sequences analysed. Homologues of the selected protein sequences were identified by blastp searches of the NCBI database (https://blast.ncbi.nlm.nih.gov/Blast.cgi) for use in phylogenetic analysis. The sequences of the top 100 protein hits were downloaded and used to construct a phylogenetic tree with the neighbour‐joining (NJ) method in MEGA 11 with 1000 bootstrap replicates, the Poisson model and pairwise deletions. The resulting tree was visualized using the iTOL website (https://itol.embl.de/).

To identify IQD proteins encoded in the pepper genome, local HMMER 3.3.2 and BLAST 2.5.0 were used with the hmm file of the IQ calmodulin‐binding motif (PFAM PF00612) and previously reported IQD protein sequences from Arabidopsis as input files (Bürstenbinder *et al*., 2017) to search for homologous proteins in the Zhangshu Harbour Pepper Database (http://ted.bti.cornell.edu/cgi‐bin/pepper/index) (*E* < 1 × 10^−5^). The candidate pepper IQD proteins were compared with the NCBI conserved domain database (https://www.ncbi.nlm.nih.gov/cdd) to confirm the presence of the IQ calmodulin‐binding motif. Sequence extraction and phylogenetic analyses of the candidate gene family were performed with TBtools‐II.

### RNA isolation and expression analysis

Total RNA was extracted using the SteadyPure Plant RNA Extraction Kit, and first‐strand cDNA was synthesized using the HiScript II 1st Strand cDNA Synthesis Kit (Vazyme). According to the manufacturer's instructions, ChamQ Universal SYBR qPCR Master Mix (Vazyme) was used to perform real‐time polymerase chain reaction on a fluorescent quantitative gene thermal cycler. The housekeeping gene *Actin‐7* (*Capana04g001698*/*Solyc11g005330.1*) was used as an internal reference. All primers used are listed in Table S1.

### Construction of transgenic overexpression and gene‐silenced lines

To construct vectors for virus‐induced gene silencing (VIGS), we identified the most effective regions for silencing of *CaIQD1*, *CaTRM‐like* and *CaOFP20* by submitting their full‐length sequences to the VIGS tool at the Sol Genomics website (https://vigs.solgenomics.net/). Referring to the VIGS method described previously (Wang *et al*., 2020; Zhou *et al*., 2021), the pTRV2 vector was double‐digested with EcoRI and BamHI, and the pTRV2‐C2b vector was digested with SmaI. After recombination, PCR identification and sequencing of single clone colonies were performed using the RNA2 F/R primer pair. Plants were inoculated by leaf injection using infection solution (10 mM MgCl_2_, 10 mM 2‐(N‐morpholino)ethanesulfonic acid and 200 μM acetosyringone), with the empty vector and a vector containing the *phytoene desaturase* (*PDS*) gene as controls. After inoculation, plants were placed in the dark for 24 h, then transferred to an artificial climate chamber with a 16‐h light (200 μmol/m^2^/s, 22 ± 2°C/8‐h dark, 20 ± 2°C) photoperiod and 70% relative humidity. Photobleaching of leaves in the positive control plants indicated successful inoculation. Positive VIGS plants were identified by PCR of new leaf tissue using the TRV virus primers RNA1 F/R and RNA2 F/R. The silencing efficiency of positive plants was evaluated by real‐time fluorescence quantitative PCR, and plant phenotypes were observed.

Because pepper lacks a stable and efficient genetic transformation system, we examined the effect of *CaIQD1* overexpression under the control of the 35S promoter in tomato (*Solanum lycopersicum* ‘Micro Tom’). The full‐length coding sequence of CaIQD1 was amplified from pepper D40 and inserted between the XhoI and XbaI restriction sites of the gate8 vector. The recombinant construct was transformed into the GV3101 strain of *Agrobacterium tumefaciens* after sequencing confirmation. Positive colonies were identified and confirmed by PCR using the 35SF/Gate8R primer pair (Supplementary Table 1) and transformed into tomato using *Agrobacterium*‐mediated genetic transformation. The vector transformation was assessed via spectinomycin, and the plant selection marker was kanamycin. Positive transgenic tomato plants were obtained by PCR identification using NPTIIF/R primers, and the efficiency of *CaIQD1* overexpression was evaluated by RT‐qPCR.

### Phenotypic characterization

Ovary morphology of the parents was observed at different developmental stages using a stereo fluorescence microscope (Leica M205 FCA). Fruit morphology was evaluated using 12 fruits from each of the four silencing‐positive *CaIQD1*, *CaTRM‐like*, *CaOFP20* lines and 27 fruits from similar node locations in the three *CaIQD1*‐overexpression lines. Fruit samples for histological observation were obtained from the same fruit part at the same developmental stage. The size and embedding direction of the samples were consistent. Safranin and fast green staining were used for observation of longitudinal paraffin sections.

### Yeast two‐hybrid (Y2H) assays

The cDNAs of *CaIQD1*, *CaOFP20*, Ca*IQD3*, Ca*SUN* and Ca*IQD17* were individually cloned into the Y2H bait vector pGBKT7. After the bait vector was confirmed to exhibit no toxicity or self‐activation, the cDNAs of *CaTRM‐like*, *CaOFP20*, Ca*KLCR1*, Ca*MAP70‐2*, Ca*CAM7*, Ca*DOF1.4*, Ca*IQD3*, Ca*SUN* and Ca*IQD17* were cloned into the Y2H prey vector pGADT7 for Y2H screening using GAL4. The designated bait and prey constructs were co‐transformed into the yeast strain Y2H Gold, and the co‐transformed yeast clones were cultured on selective media.

### Luciferase complementation imaging (LCI) assay

The coding sequences of *CaIQD1*, *CaTRM‐like*, *CaOFP20*, Ca*KLCR1*, Ca*MAP70‐2*, Ca*CAM7*, Ca*DOF1.4*, Ca*IQD3*, Ca*SUN* and Ca*IQD17* were cloned into the pCAMBIA1300‐nLUC and pCAMBIA1300‐cLUC vectors and transformed into *Agrobacterium tumefaciens* strain GV3101. *Agrobacterium* strains containing the designated plasmids and p19 plasmids (to suppress gene silencing) were co‐infiltrated into leaves of *Nicotiana benthamiana* for transient expression. After 48 h, D‐luciferin potassium salt was sprayed onto the backs of tobacco leaves, and Lumozen was used to capture luminescence images. The primers used for all constructs are listed in Table S1.

### Pull‐down assay

The coding sequence of CaIQD1 was cloned into the pGEX‐6P‐1 vector and transformed into BL21 (DE3) competent cells to produce the GST‐CaIQD1 fusion protein. The recombinant vectors of CaOFP20 (PET‐B2M), CaCAM7 (pet‐sumo), CaKLCR1 (PET22B‐NEXT) and CaDOF1.4 (PET22B‐NEXT) were transformed into BL21 (DE3) competent cells to produce HIS‐CaOFP20, HIS‐CaCAM7, HIS‐CaKLCR1 and HIS‐DOF1.4 fusion proteins. For GST pull‐down analysis, 500 μg recombinant purified GST‐CaIQD1 or GST was incubated with glutathione–agarose beads at 25°C for 2 h, and then, 500 μg single HIS‐labelled fusion protein was added. After incubation for 16 h, the beads were washed three times to remove the unbound proteins, then boiled in 1× SDS sample buffer to elute the bound proteins. The proteins were separated by gel electrophoresis, and western blotting was performed with the appropriate antibodies.

### Co‐IP assay

The coding sequences of *CaIQD1* and *CaMAP70‐2* were used to construct pCAMBIA1300‐35S‐CaIQD1‐HA and pCAMBIA1300‐35S‐CaMAP70‐2‐Myc recombinant vectors. The fusion constructs were transformed into *Agrobacterium* and co‐infiltrated into *N. benthamiana* leaves. After 72 h of culture, the leaves were harvested and ground in liquid nitrogen, and total protein was extracted by adding protein extraction solution (20 mM Tris–HCl, 5 mM EDTA, 150 mM NaCl, 0.25% NP‐40 and 1× protease inhibitor cocktail) at a ratio of 1 g/3 mL. Anti‐HA magnetic beads were incubated at 4°C for 2 h and then washed with buffer. The immunoprecipitates were electrophoresed using precast gels from GenScript Biotech Co., Ltd. (SurePAGE, Bis‐Tris, 10 × 8, 4%–20%, 12 wells) and examined by immunoblotting with anti‐HA and anti‐MYC antibodies.

### Subcellular localization of target genes

The individual fusion constructs 35S:*CaIQD1*/*CaOFP20/CaMAP70‐2/CaCAM7/CaDOF1.4/CaIQD3/CaSUN/CaIQD17‐GFP* and the control construct (35S:*GFP*) were transformed into *N. benthamiana* leaves, together with the nuclear marker *mCherry*. Transformed tobacco plants were cultured for 48 h and then examined with a confocal laser scanning microscope (LSM 710 META, Zeiss, Germany). Because 35S:*CaTRM‐like*‐*GFP* and 35S:*CaKLCR1*‐*GFP* were only weakly expressed in tobacco leaves, these constructs and the control construct (35S:*GFP*) were transformed together with *mCherry* into *N. benthamiana* protoplasts. Transformed cells were cultured for 18 h and then examined as above. When the two proteins are co‐located, different fluorescent linkers (GFP and mKate) are connected for observation.

### BiFC assay

The full‐length coding sequences of *CaIQD1* and *CaOFP20* were ligated into the BamHI restriction site of the pSPYNE‐35S vector, and the full‐length coding sequences of *CaOFP20*, *CaTRM‐like*, *CaMAP70‐2*, *CaCAM7*, *CaDOF1.4*, *CaIQD3*, *CaSUN* and *CaIQD17* were ligated into the BamHI restriction site of the pSPYCE‐35S vector. The resulting plasmids were transformed into *A. tumefaciens* strain GV3101, and leaves of *N. benthamiana* were co‐infiltrated with equal amounts of *Agrobacterium* expressing pairs of BiFC constructs, along with the p19 silencing plasmid. The fluorescence signals were examined under a confocal laser scanning microscope 2–3 days after infiltration.

### Quantification and statistical analysis

Data are presented as mean ± standard deviation (SD). The significance of differences was evaluated using two‐tailed Student's *t*‐tests. Numbers of replicates are indicated in the figure legends.

## Conflicts of interest

The authors declare no competing interests.

## Author contributions

Z.L. provided the material. L.M., L.O., X.Z. and Z.L. co‐designed the study and drafted the manuscript. L.M. and Y.S. did most of the molecular experiments. L.M., L.O., X.Z. and Z.L. modified the manuscript. Q.C. and Y.H. participated in the construction, investigation and cloning of genetic populations. X.Z. carried out the relevant bioinformatics analysis. J.L. participated in the experimental data analysis. X.Z., J.L., W.X., D.Z., N.F., D.C., Z.W. and P.L. participated in the experiment. M.D. provided the necessary technical guidance and advice. All authors reviewed and approved the manuscript for publication.

## Supporting information


**Figure S1** Preliminary positioning intervals for parental phenotypes and fruit shape traits.
**Figure S2** Phylogenetic analysis of CaIQD1 and phenotypic indicators of functional validation plants.
**Figure S3** Yeast two‐hybrid test verified the interaction between CaIQD1, CaOFP20, and CaTRM‐like protein.
**Figure S4** Analysis of *CaTRM‐like* and *CaOFP20*‐silenced plant lines.
**Figure S5** Verification of the interaction between CaOFP20 and three IQD proteins and phylogenetic tree analysis of the entire IQD family of pepper proteins.
**Figure S6** Expression of microtubule‐associated genes in TRV: *CaIQD1* and 35S: *CaIQD1*.
**Figure S7** Analysis of the differences between CaIQD1 and CaSUN/CaIQD17/CaIQD3.
**Figure S8** Co‐location analysis of CaKLCR1 and CaIQD1/CaIQD17/CaSUN.

**Table S1** Primers used in this study.
**Table S2** A loc file for QTL mapping analysis.
**Table S3** A map file for QTL mapping analysis.
**Table S4** A F_2_.qua file for QTL mapping analysis.
**Table S5** Result of linkage analysis with molecular markers by MapQTL4.0.
**Table S6** Statistics of genotyping results of F_3_ recombinant individual plants.
**Table S7** Detailed information of SNP‐index values and variations for FSI in the 649‐kb candidate interval.

## Data Availability

The data that supports the findings of this study are available in the supplementary material of this article.
