# Supplementary material for: The IQ67‐domain protein IQD1 regulates fruit shape through complex multiprotein interactions in pepper (Capsicum annuum L.)
Source: Plant Biotechnol J. 2025 Apr 11;23(7):2651–66. doi: 10.1111/pbi.70078 (PMC12205865; doi:10.1111/pbi.70078)
Supplement: Supplementary file 1 — Figure S1 Preliminary positioning intervals for parental phenotypes and fruit shape traits. Figure S2 Phylogenetic analysis of CaIQD1 and phenotypic indicators of functional validation plants. Figure S3 Yeast two‐hybrid test verified the interaction between CaIQD1, CaOFP20, and CaTRM‐like protein. Figure S4 Analysis of CaTRM‐like and CaOFP20‐silenced plant lines. Figure S5 Verification of the interaction between CaOFP20 and three IQD proteins and phylogenetic tree analysis of the entire IQD family of pepper proteins. Figure S6 Expression of microtubule‐associated genes in TRV: CaIQD1 and 35S: CaIQD1. Figure S7 Analysis of the differences between CaIQD1 and CaSUN/CaIQD17/CaIQD3. Figure S8 Co‐location analysis of CaKLCR1 and CaIQD1/CaIQD17/CaSUN. [file PBI-23-2651-s002.zip › Supplementary Figures.pdf]

The IQ67-domain protein IQD1 regulates fruit shape through complex multi-protein interactions in pepper (*Capsicum annuum* L.)

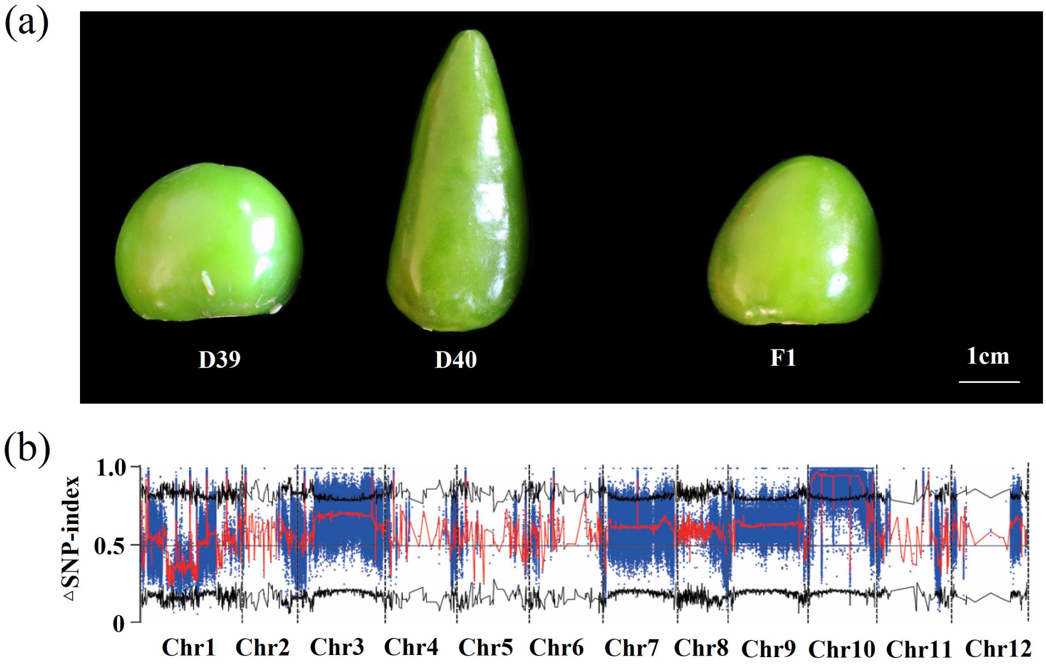

Figure S1 Preliminary positioning intervals for parental phenotypes and fruit shape traits. (a) Phenotypes of parents and F_1. (b) SNP distribution Δ(SNP index) plot.

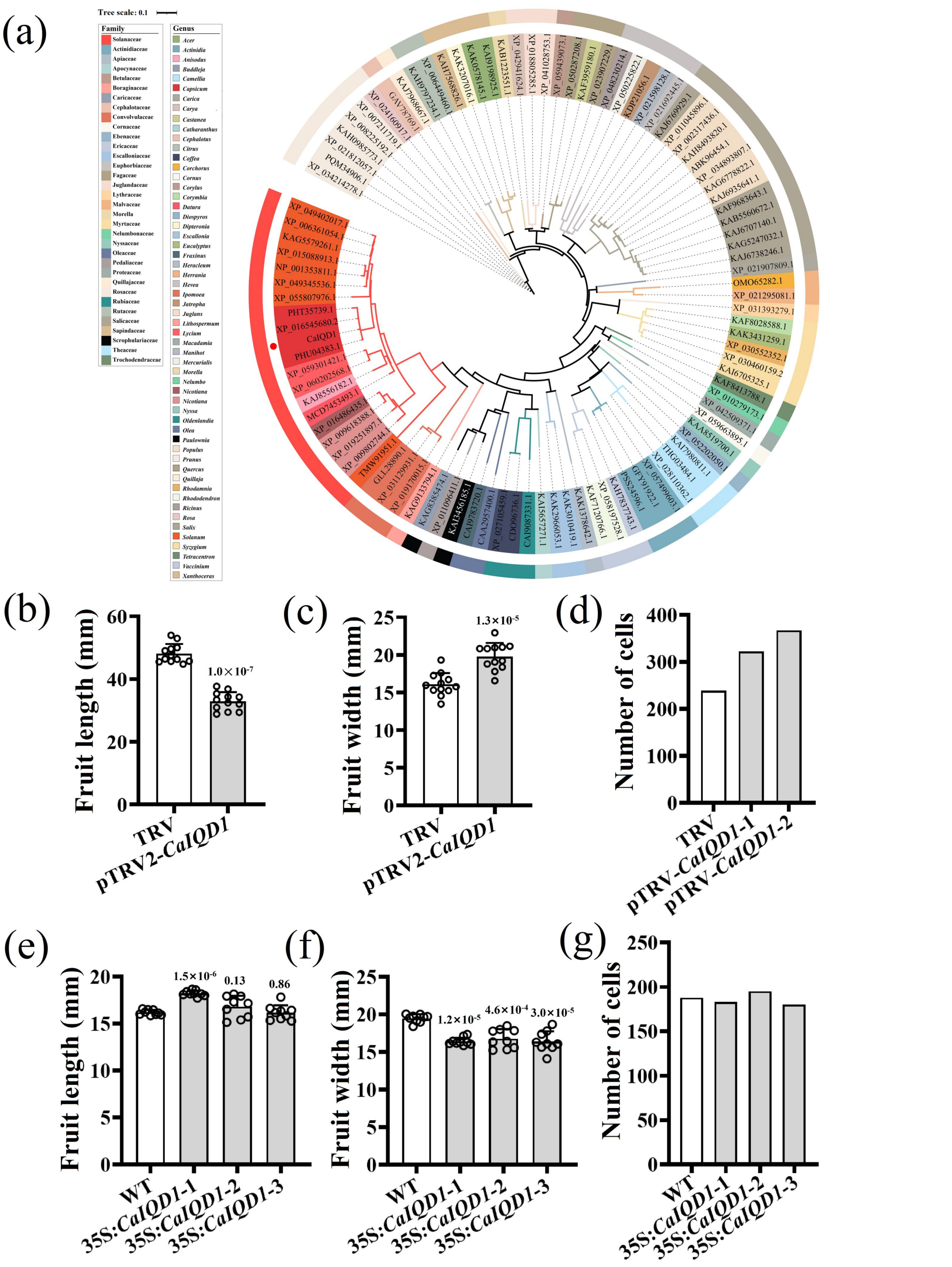

Figure S2 Phylogenetic analysis of CaIQD1 and phenotypic indicators of functional validation plants. (a) Phylogenetic analysis of CaIQD1. Genes of species belonging to the same family and genus are marked with the same color. The outer circle represents the family and the inner circle represents the genus. In the terminology of the gene name, the letter before the dash is the species code, and the letter after the dash is the gene name. CaIQD1 is indicated by red dots. (b, c) The fruit length and width in TRV: *CaIQD1* fruits. Data come from 12 fruits on four silent *CaIQD1* lines. (d) Total cell number of longitudinal sections of TRV and TRV: *CaIQD1* fruits. (e-f) The fruit length and width in 35S: *CaIQD1* fruits. Data come from three over-expressive *CaIQD1* lines. (g) Total cell number of longitudinal sections of WT and 35S: *CaIQD1* fruits. The significance of differences was evaluated using two-tailed Student's t-tests, with exact P values indicated on the graph.

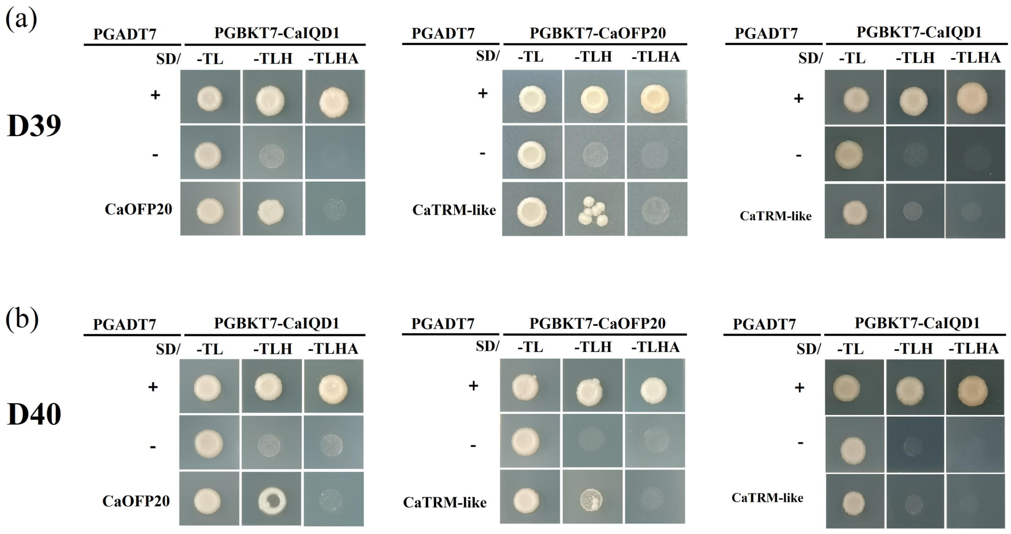

Figure S3. Yeast two-hybrid test verified the interaction between CaIQD1, CaOFP20, and CaTRM-like protein.(a) Yeast two-hybrid assay was used to detect the interaction between CaIQD1, CaOFP20, and CaTRM-like protein in D39. (b) Yeast two-hybrid assay was used to detect the interaction between CaIQD1, CaOFP20, and CaTRM-like protein in D40.

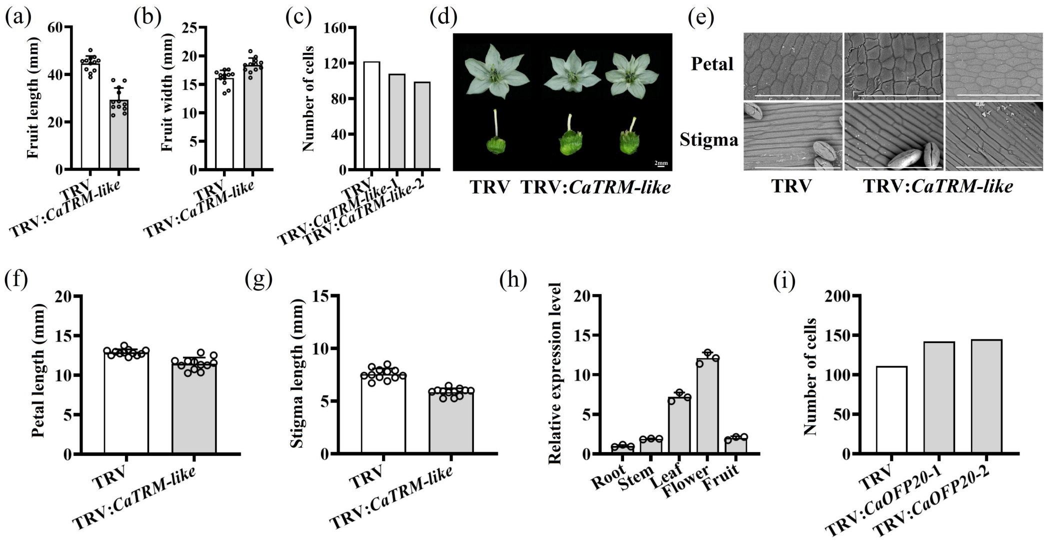

Figure S4 Analysis of *CaTRM-like* and *CaOFP20* silenced plant lines. (a-b) The fruit length and width in TRV: *CaTRM-like* fruits. Data come from 12 fruits on four silent *CaTRM-like lines*. (c) Total cell number of longitudinal sections of TRV and TRV: *CaTRM-like* fruits. (d) Flower morphology of pepper after silent *CaTRM-like*. Scale bar = 2cm. (e) Scanning electron microscope (SEM) of flowers and stigmas after silencing *CaTRM-like*. Scale bar = 200μm (petals), 100μm (stigma). (f-g) The petal and stigma length in TRV: *CaTRM-like* plants. (h) The expression characteristics of *CaTRM-like* in different tissues of D40. (i) Total cell number of TRV and TRV: *CaOFP20* fruits.

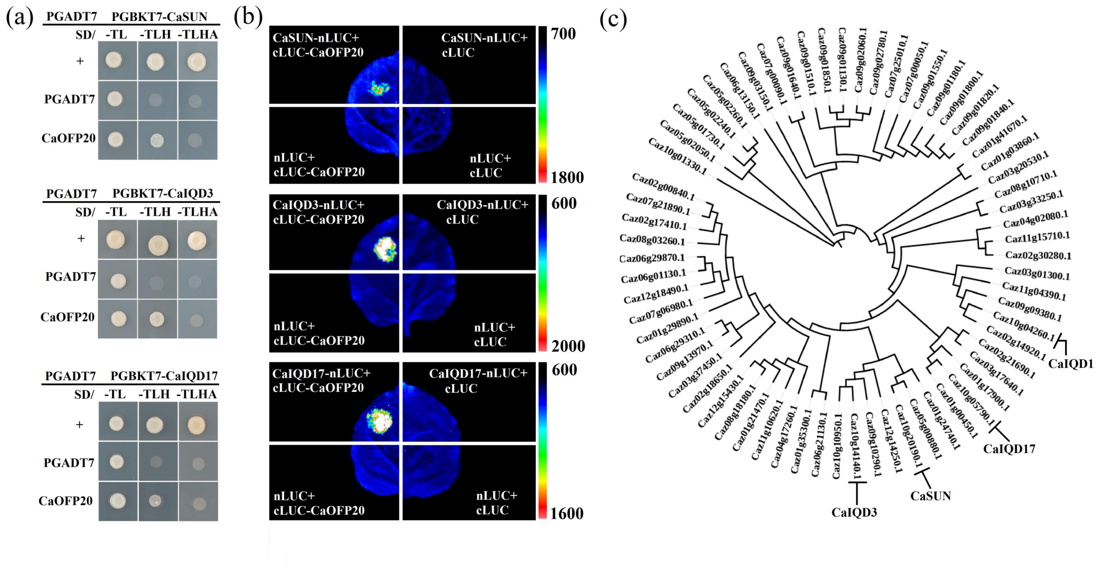

Figure S5 Verification of the interaction between CaOFP20 and three IQD proteins and phylogenetic tree analysis of the entire IQD family of pepper proteins. (a) Yeast two-hybrid assay was used to detect the interaction between CaOFP20 and CaSUN, CaIQD3, and CaIQD17. (b) The interaction between CaOFP20 and CaSUN, CaIQD3, and CaIQD17 was verified in vivo by mitotic luciferase assay. (c) Evolutionary tree analysis of IQD family proteins in pepper.

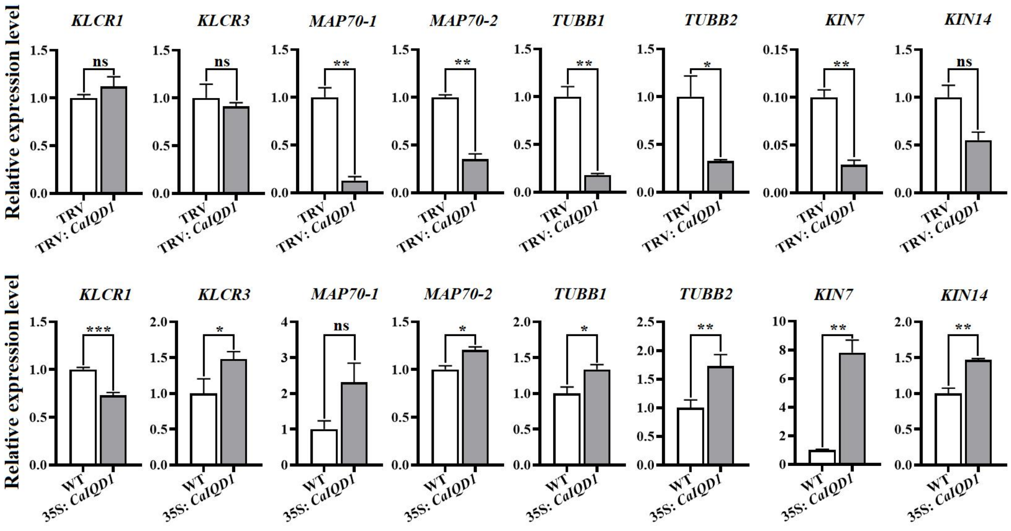

Figure S6 Expression of microtubule-associated genes in TRV: *CaIQD1* and 35S: *CaIQD1*. Note: Two-tailed Student 't-test was used to evaluate the significance of the difference. "*" means p<0.05, "**" means p<0.01, and "***" means p<0.001.

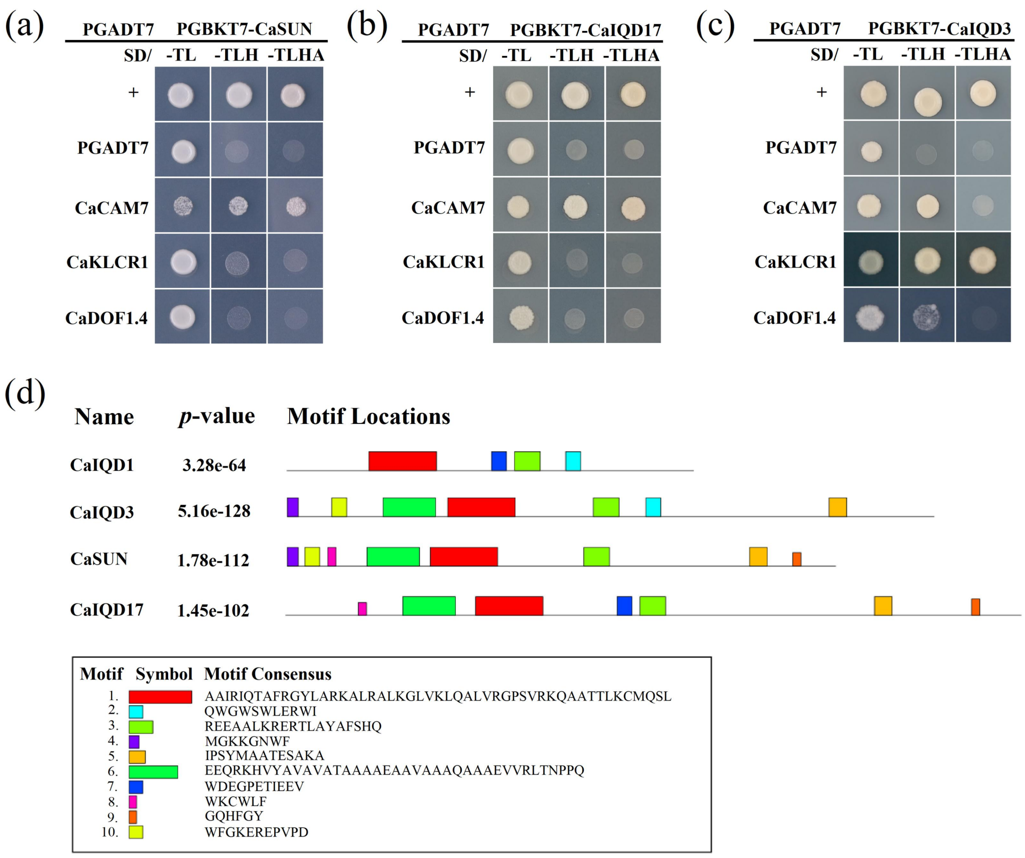

Figure S7 Analysis of the differences between CaIQD1 and CaSUN/CaIQD17/CaIQD3. (a) Y2H assays revealed interactions between CaSUN and CaCAM7/CaKLCR1/CaDOF1.4. (b) Y2H assays revealed interactions between CaIQD17 and CaCAM7/CaKLCR1/CaDOF1.4. (c) Y2H assays revealed interactions between CaIQD3 and CaCAM7/CaKLCR1/CaDOF1.4. (d) Motif analysis of CaIQD1, CaIQD3, CaSUN， and CaIQD17.

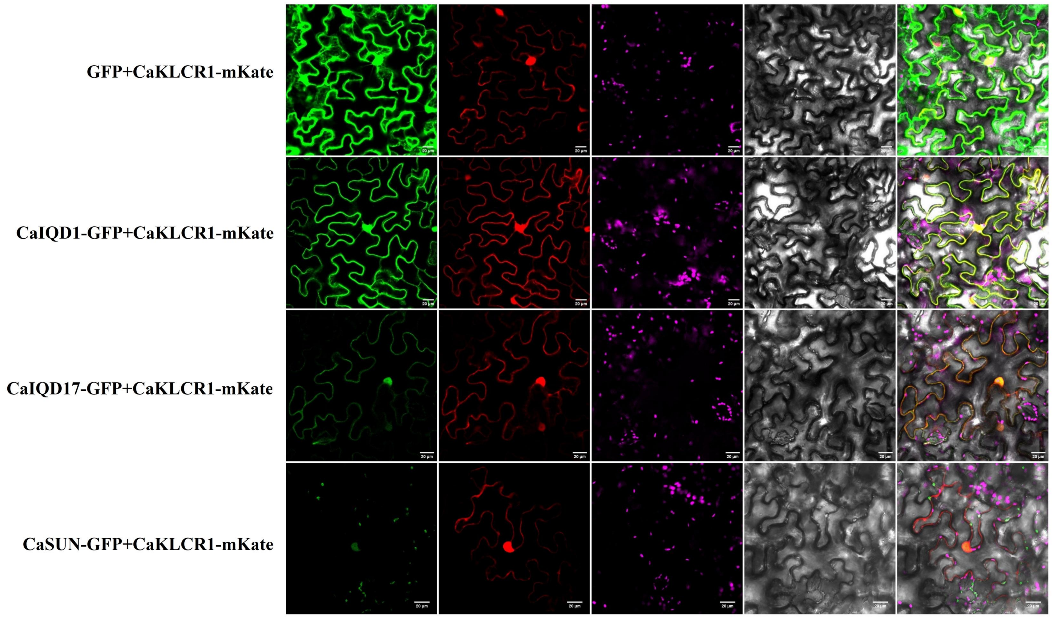

Figure S8 Co-location analysis of CaKLCR1 and CaIQD1/ CaIQD17/ CaSUN. From left to right are Protein-GFP, Protein-mKate, Chloroplast autofluorescence, Bright field, and Overlay. Scale bar = 20 μm.
