# Supplementary figures and images for: The IQ67‐domain protein IQD1 regulates fruit shape through complex multiprotein interactions in pepper (Capsicum annuum L.)
Source: Plant Biotechnol J. 2025 Apr 11;23(7):2651–66. doi: 10.1111/pbi.70078 (PMC12205865; doi:10.1111/pbi.70078)

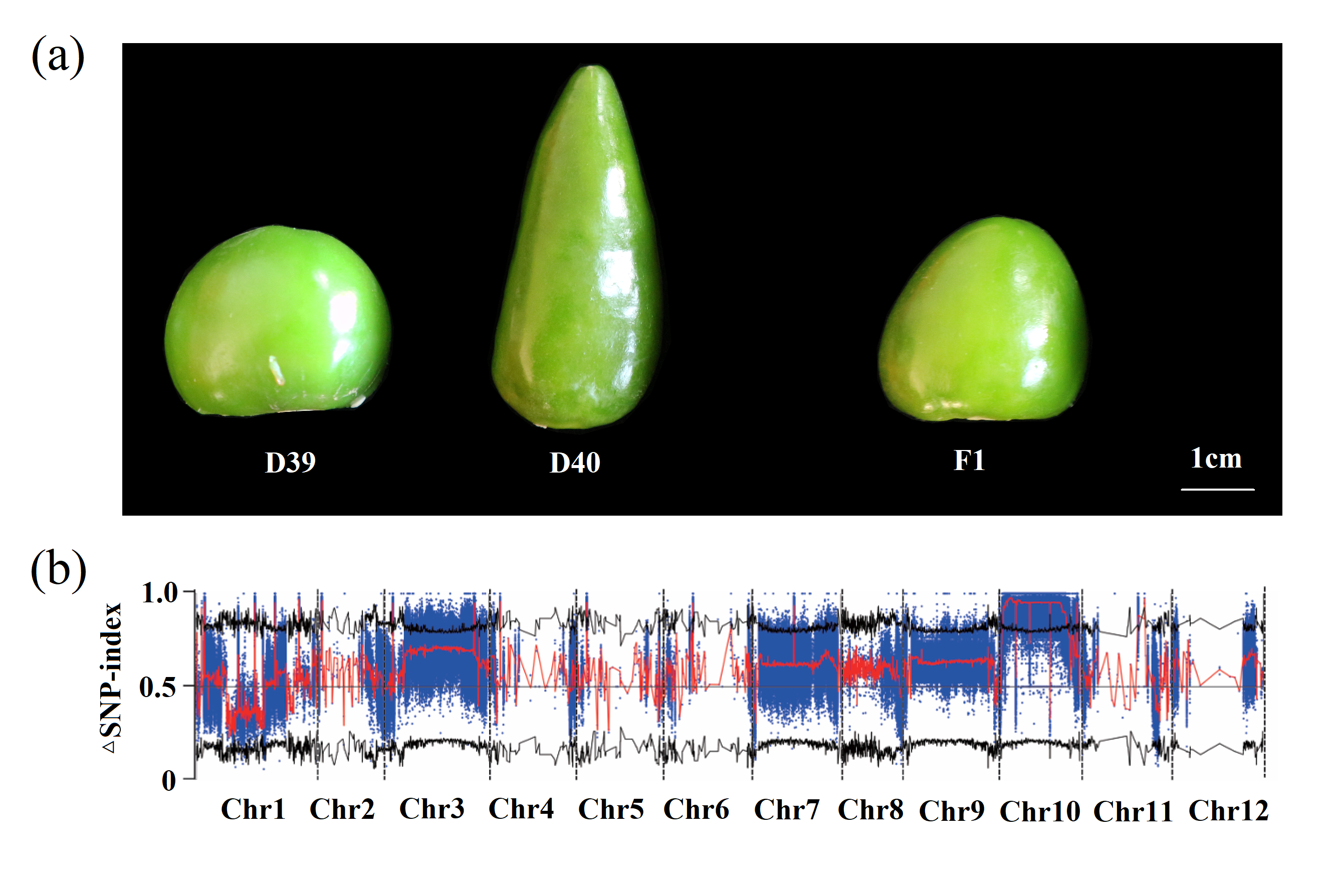

Supplement: Supplementary file 1 — Figure S1 Preliminary positioning intervals for parental phenotypes and fruit shape traits. Figure S2 Phylogenetic analysis of CaIQD1 and phenotypic indicators of functional validation plants. Figure S3 Yeast two‐hybrid test verified the interaction between CaIQD1, CaOFP20, and CaTRM‐like protein. Figure S4 Analysis of CaTRM‐like and CaOFP20‐silenced plant lines. Figure S5 Verification of the interaction between CaOFP20 and three IQD proteins and phylogenetic tree analysis of the entire IQD family of pepper proteins. Figure S6 Expression of microtubule‐associated genes in TRV: CaIQD1 and 35S: CaIQD1. Figure S7 Analysis of the differences between CaIQD1 and CaSUN/CaIQD17/CaIQD3. Figure S8 Co‐location analysis of CaKLCR1 and CaIQD1/CaIQD17/CaSUN. [file PBI-23-2651-s002.zip › PBI_70078_f1_Figure S1_2.tiff]

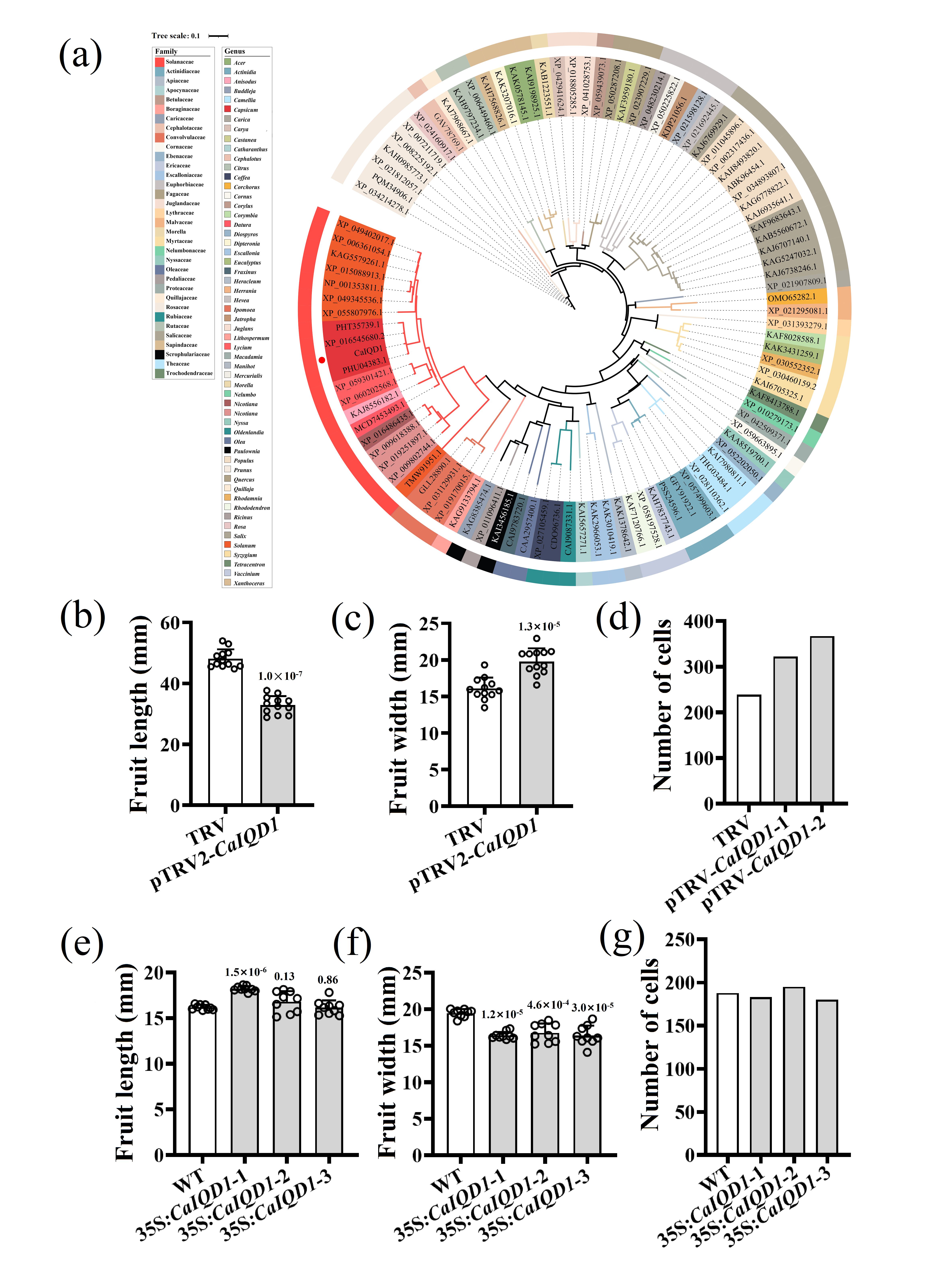

Supplement: Supplementary file 1 — Figure S1 Preliminary positioning intervals for parental phenotypes and fruit shape traits. Figure S2 Phylogenetic analysis of CaIQD1 and phenotypic indicators of functional validation plants. Figure S3 Yeast two‐hybrid test verified the interaction between CaIQD1, CaOFP20, and CaTRM‐like protein. Figure S4 Analysis of CaTRM‐like and CaOFP20‐silenced plant lines. Figure S5 Verification of the interaction between CaOFP20 and three IQD proteins and phylogenetic tree analysis of the entire IQD family of pepper proteins. Figure S6 Expression of microtubule‐associated genes in TRV: CaIQD1 and 35S: CaIQD1. Figure S7 Analysis of the differences between CaIQD1 and CaSUN/CaIQD17/CaIQD3. Figure S8 Co‐location analysis of CaKLCR1 and CaIQD1/CaIQD17/CaSUN. [file PBI-23-2651-s002.zip › PBI_70078_f2_Figure S2_2.tiff]

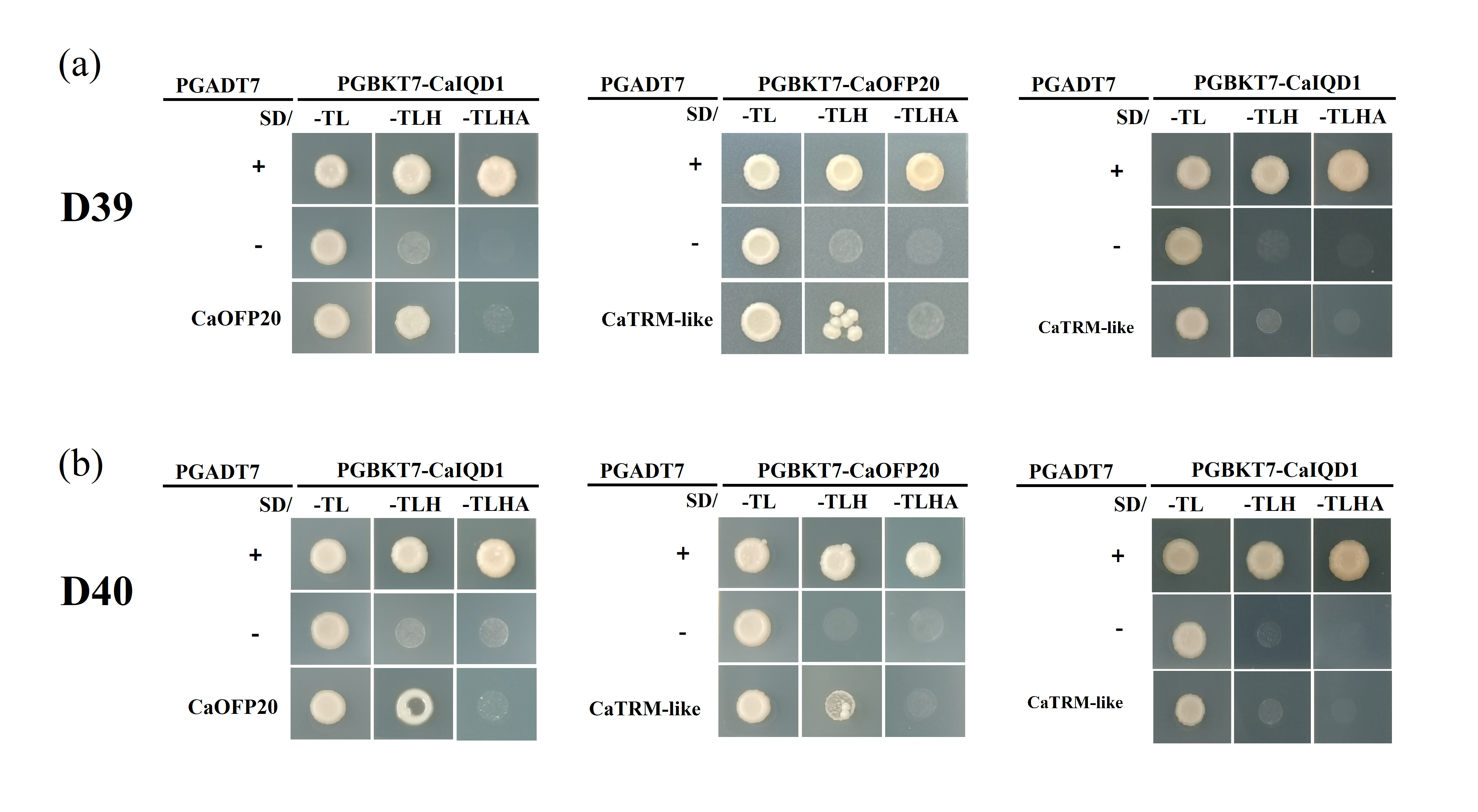

Supplement: Supplementary file 1 — Figure S1 Preliminary positioning intervals for parental phenotypes and fruit shape traits. Figure S2 Phylogenetic analysis of CaIQD1 and phenotypic indicators of functional validation plants. Figure S3 Yeast two‐hybrid test verified the interaction between CaIQD1, CaOFP20, and CaTRM‐like protein. Figure S4 Analysis of CaTRM‐like and CaOFP20‐silenced plant lines. Figure S5 Verification of the interaction between CaOFP20 and three IQD proteins and phylogenetic tree analysis of the entire IQD family of pepper proteins. Figure S6 Expression of microtubule‐associated genes in TRV: CaIQD1 and 35S: CaIQD1. Figure S7 Analysis of the differences between CaIQD1 and CaSUN/CaIQD17/CaIQD3. Figure S8 Co‐location analysis of CaKLCR1 and CaIQD1/CaIQD17/CaSUN. [file PBI-23-2651-s002.zip › PBI_70078_f3_Figure S3_2.tif]

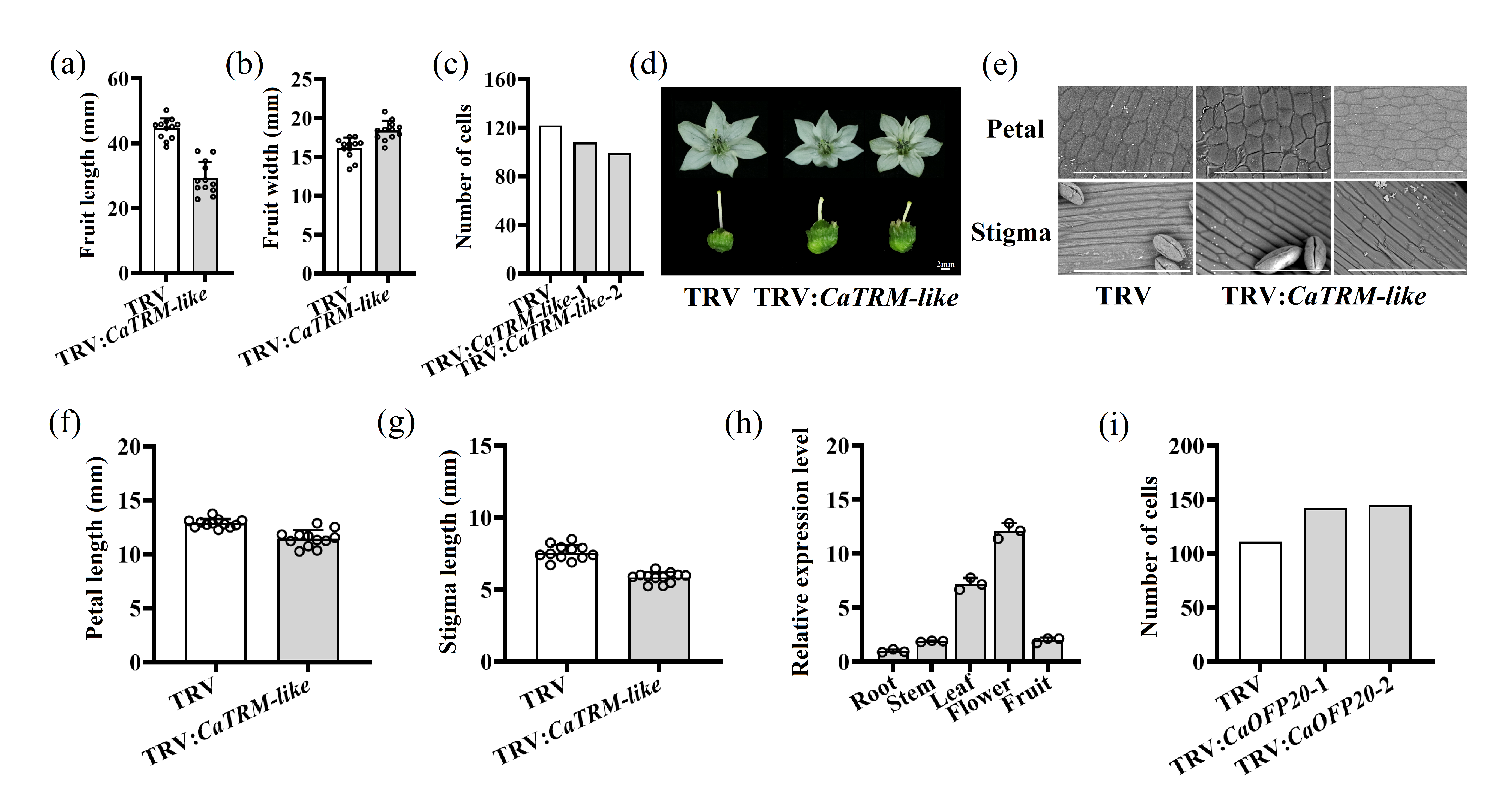

Supplement: Supplementary file 1 — Figure S1 Preliminary positioning intervals for parental phenotypes and fruit shape traits. Figure S2 Phylogenetic analysis of CaIQD1 and phenotypic indicators of functional validation plants. Figure S3 Yeast two‐hybrid test verified the interaction between CaIQD1, CaOFP20, and CaTRM‐like protein. Figure S4 Analysis of CaTRM‐like and CaOFP20‐silenced plant lines. Figure S5 Verification of the interaction between CaOFP20 and three IQD proteins and phylogenetic tree analysis of the entire IQD family of pepper proteins. Figure S6 Expression of microtubule‐associated genes in TRV: CaIQD1 and 35S: CaIQD1. Figure S7 Analysis of the differences between CaIQD1 and CaSUN/CaIQD17/CaIQD3. Figure S8 Co‐location analysis of CaKLCR1 and CaIQD1/CaIQD17/CaSUN. [file PBI-23-2651-s002.zip › PBI_70078_f4_Figure S4_2.tiff]

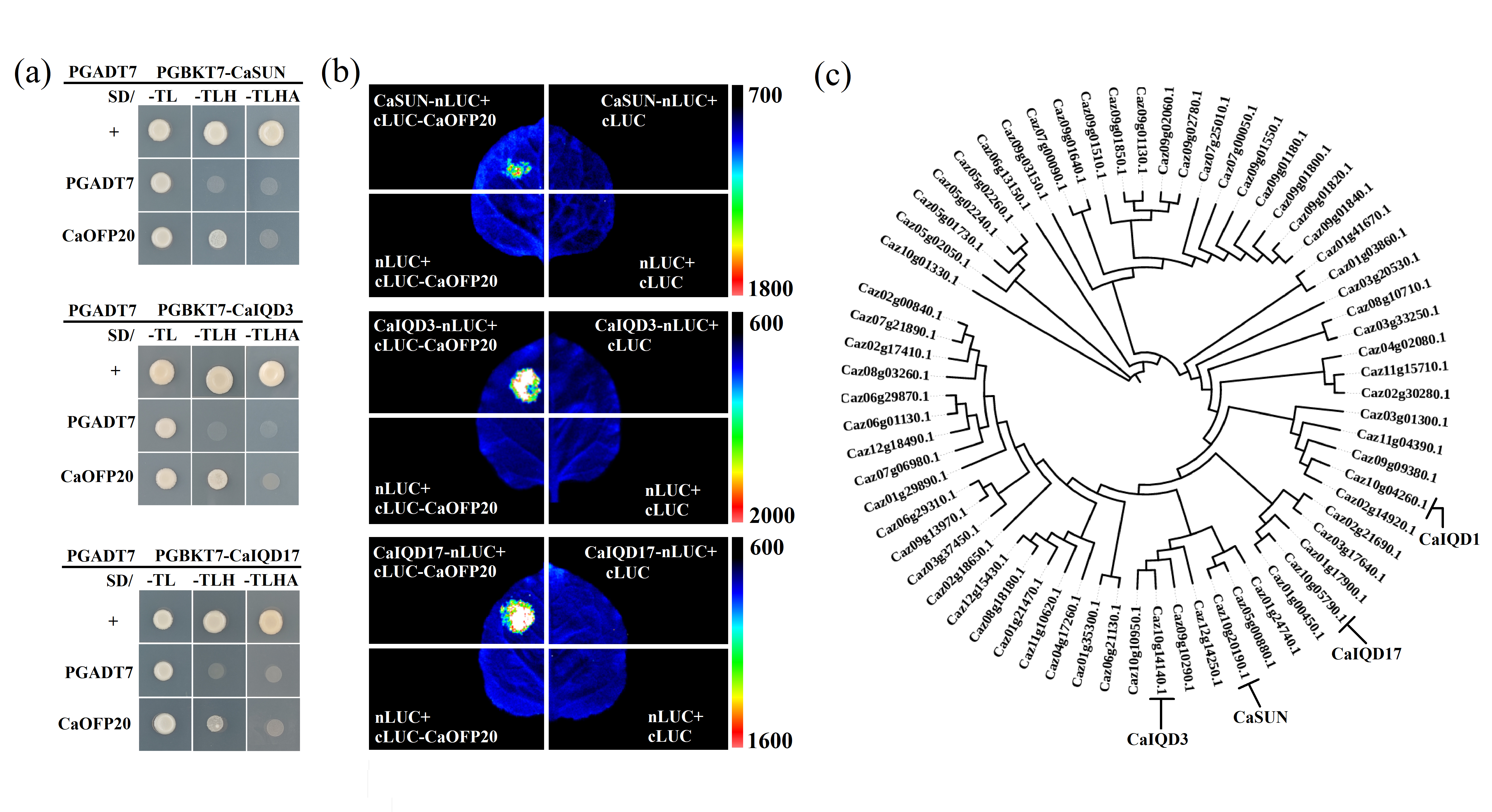

Supplement: Supplementary file 1 — Figure S1 Preliminary positioning intervals for parental phenotypes and fruit shape traits. Figure S2 Phylogenetic analysis of CaIQD1 and phenotypic indicators of functional validation plants. Figure S3 Yeast two‐hybrid test verified the interaction between CaIQD1, CaOFP20, and CaTRM‐like protein. Figure S4 Analysis of CaTRM‐like and CaOFP20‐silenced plant lines. Figure S5 Verification of the interaction between CaOFP20 and three IQD proteins and phylogenetic tree analysis of the entire IQD family of pepper proteins. Figure S6 Expression of microtubule‐associated genes in TRV: CaIQD1 and 35S: CaIQD1. Figure S7 Analysis of the differences between CaIQD1 and CaSUN/CaIQD17/CaIQD3. Figure S8 Co‐location analysis of CaKLCR1 and CaIQD1/CaIQD17/CaSUN. [file PBI-23-2651-s002.zip › PBI_70078_f5_Figure S5_2.tiff]

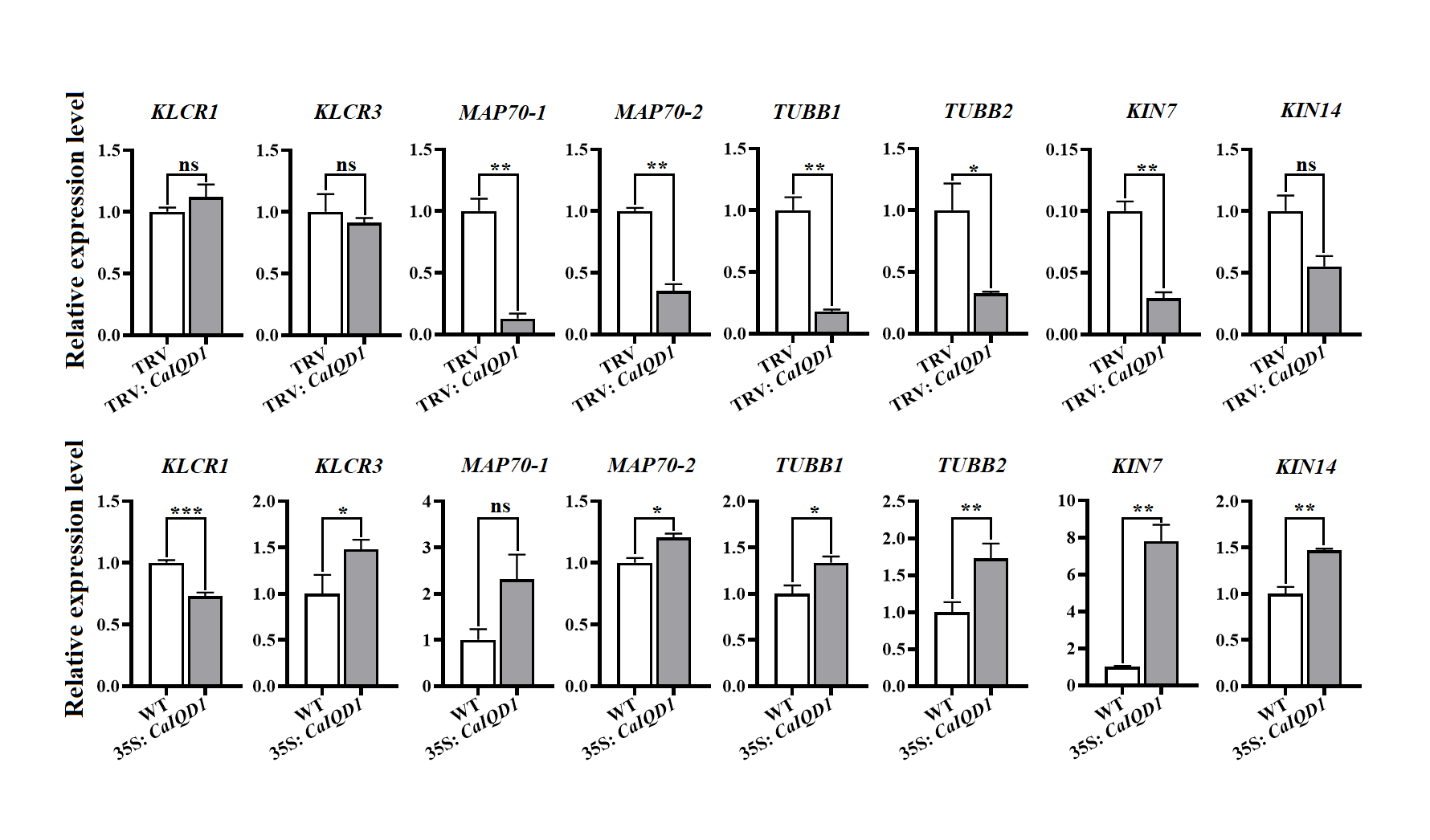

Supplement: Supplementary file 1 — Figure S1 Preliminary positioning intervals for parental phenotypes and fruit shape traits. Figure S2 Phylogenetic analysis of CaIQD1 and phenotypic indicators of functional validation plants. Figure S3 Yeast two‐hybrid test verified the interaction between CaIQD1, CaOFP20, and CaTRM‐like protein. Figure S4 Analysis of CaTRM‐like and CaOFP20‐silenced plant lines. Figure S5 Verification of the interaction between CaOFP20 and three IQD proteins and phylogenetic tree analysis of the entire IQD family of pepper proteins. Figure S6 Expression of microtubule‐associated genes in TRV: CaIQD1 and 35S: CaIQD1. Figure S7 Analysis of the differences between CaIQD1 and CaSUN/CaIQD17/CaIQD3. Figure S8 Co‐location analysis of CaKLCR1 and CaIQD1/CaIQD17/CaSUN. [file PBI-23-2651-s002.zip › PBI_70078_f6_Figure S6_2.tif]

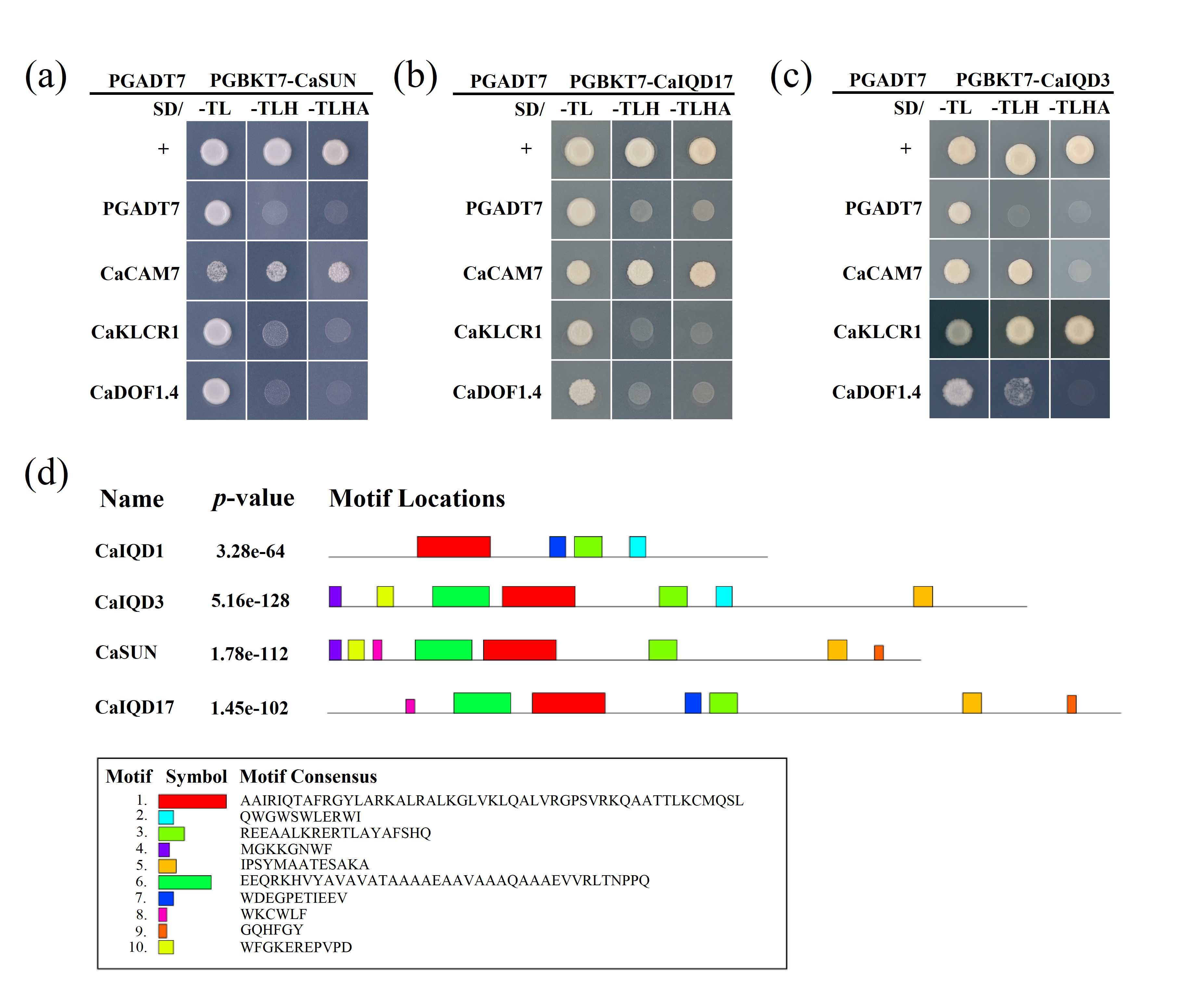

Supplement: Supplementary file 1 — Figure S1 Preliminary positioning intervals for parental phenotypes and fruit shape traits. Figure S2 Phylogenetic analysis of CaIQD1 and phenotypic indicators of functional validation plants. Figure S3 Yeast two‐hybrid test verified the interaction between CaIQD1, CaOFP20, and CaTRM‐like protein. Figure S4 Analysis of CaTRM‐like and CaOFP20‐silenced plant lines. Figure S5 Verification of the interaction between CaOFP20 and three IQD proteins and phylogenetic tree analysis of the entire IQD family of pepper proteins. Figure S6 Expression of microtubule‐associated genes in TRV: CaIQD1 and 35S: CaIQD1. Figure S7 Analysis of the differences between CaIQD1 and CaSUN/CaIQD17/CaIQD3. Figure S8 Co‐location analysis of CaKLCR1 and CaIQD1/CaIQD17/CaSUN. [file PBI-23-2651-s002.zip › PBI_70078_f7_Figure S7_2.tiff]

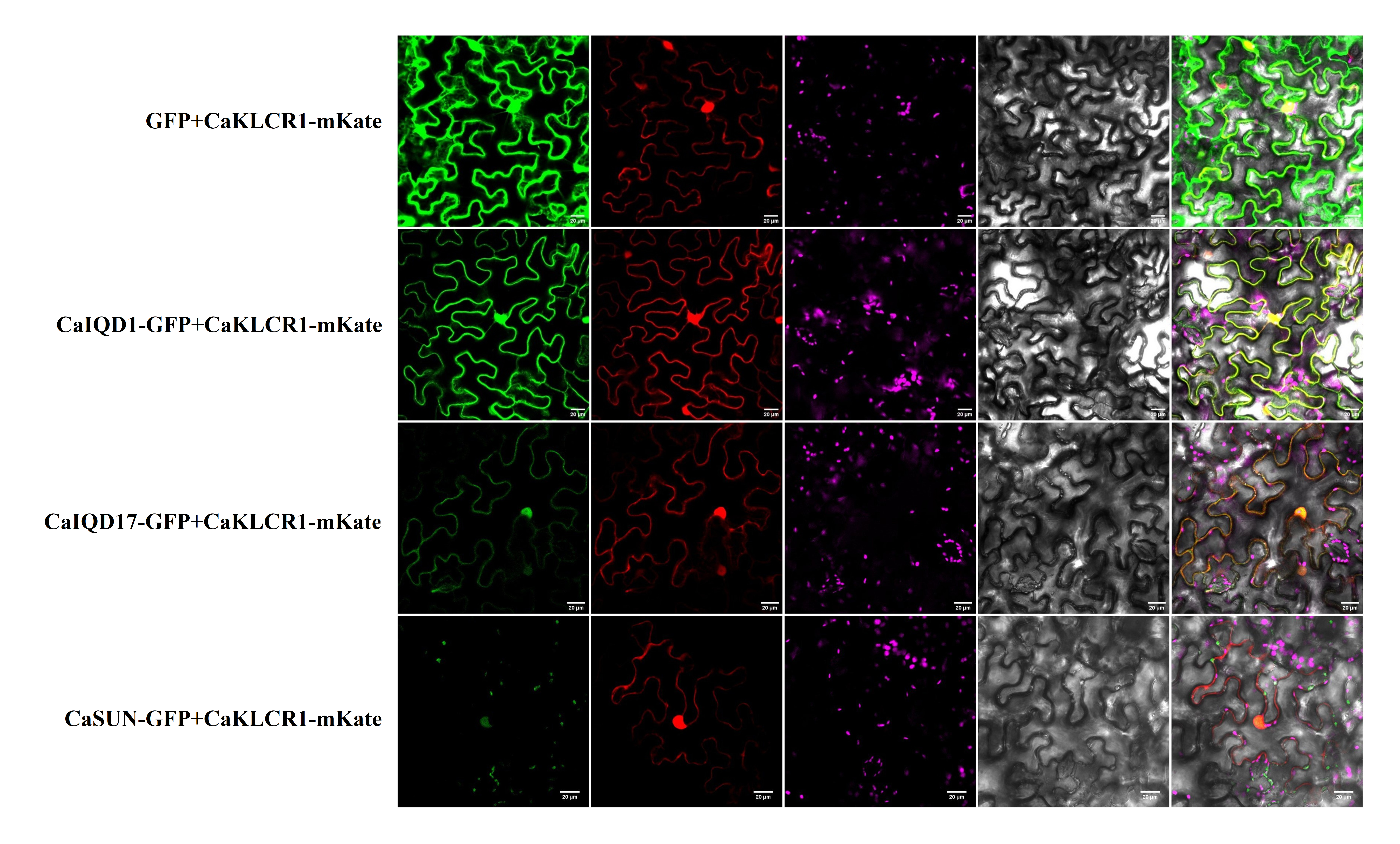

Supplement: Supplementary file 1 — Figure S1 Preliminary positioning intervals for parental phenotypes and fruit shape traits. Figure S2 Phylogenetic analysis of CaIQD1 and phenotypic indicators of functional validation plants. Figure S3 Yeast two‐hybrid test verified the interaction between CaIQD1, CaOFP20, and CaTRM‐like protein. Figure S4 Analysis of CaTRM‐like and CaOFP20‐silenced plant lines. Figure S5 Verification of the interaction between CaOFP20 and three IQD proteins and phylogenetic tree analysis of the entire IQD family of pepper proteins. Figure S6 Expression of microtubule‐associated genes in TRV: CaIQD1 and 35S: CaIQD1. Figure S7 Analysis of the differences between CaIQD1 and CaSUN/CaIQD17/CaIQD3. Figure S8 Co‐location analysis of CaKLCR1 and CaIQD1/CaIQD17/CaSUN. [file PBI-23-2651-s002.zip › PBI_70078_f8_Figure S8_2.tiff]
